# Coding variants in NOD-like receptors: An association study on risk and survival of colorectal cancer

**DOI:** 10.1371/journal.pone.0199350

**Published:** 2018-06-21

**Authors:** Stefanie Huhn, Miguel I. da Silva Filho, Tharmila Sanmuganantham, Tica Pichulik, Calogerina Catalano, Barbara Pardini, Alessio Naccarati, Veronika Polakova-Vymetálkova, Katerina Jiraskova, Ludmila Vodickova, Pavel Vodicka, Markus W. Löffler, Lioba Courth, Jan Wehkamp, Farhat V. N. Din, Maria Timofeeva, Susan M. Farrington, Lina Jansen, Kari Hemminki, Jenny Chang-Claude, Hermann Brenner, Michael Hoffmeister, Malcolm G. Dunlop, Alexander N. R. Weber, Asta Försti

**Affiliations:** 1 Department of Molecular Genetic Epidemiology, German Cancer Research Center (DKFZ), Heidelberg, Germany; 2 Department of Multiple Myeloma, Internal Medicine V: Hematology, Oncology and Rheumatology, Heidelberg University Hospital, Heidelberg, Germany; 3 Interfaculty Institute for Cell Biology, Department of Immunology, University of Tübingen, Tübingen, Germany; 4 Italian Institute for Genomic Medicine (IIGM), Turin, Italy; 5 Institute of Experimental Medicine, Academy of Sciences of the Czech Republic, Prague, Czech Republic; 6 Institute of Biology and Medical Genetics, 1st Faculty of Medicine, Charles University, Prague, Czech Republic; 7 Biomedical Centre, Faculty of Medicine Pilsen, Charles University Prague, Pilsen, Czech Republic; 8 Department of General, Visceral and Transplant Surgery, University Hospital Tübingen, Tübingen, Germany; 9 Department of Internal Medicine I, University Hospital Tübingen, Tübingen, Germany; 10 Colon Cancer Genetics Group, MRC Human Genetics Unit, The University of Edinburgh, Western General Hospital, Edinburgh, United Kingdom; 11 Division of Clinical Epidemiology and Aging Research, German Cancer Research Center (DKFZ), Heidelberg, Germany; 12 Center for Primary Health Care Research, Clinical Research Center, Lund University, Malmö, SE, Sweden; 13 Division of Cancer Epidemiology, German Cancer Research Center (DKFZ), Heidelberg, Germany; 14 Division of Preventive Oncology, German Cancer Research Center (DKFZ) and National Center for Tumor Diseases (NCT), Im Neuenheimer Feld 460, Heidelberg, Germany; 15 German Cancer Consortium (DKTK), German Cancer Research Center (DKFZ), Heidelberg, Germany; University of South Alabama Mitchell Cancer Institute, UNITED STATES

## Abstract

Nod-like receptors (NLRs) are important innate pattern recognition receptors and regulators of inflammation or play a role during development. We systematically analysed 41 non-synonymous single nucleotide polymorphisms (SNPs) in 21 NLR genes in a Czech discovery cohort of sporadic colorectal cancer (CRC) (1237 cases, 787 controls) for their association with CRC risk and survival. Five SNPs were found to be associated with CRC risk and eight with survival at 5% significance level. In a replication analysis using data of two large genome-wide association studies (GWASs) from Germany (DACHS: 1798 cases and 1810 controls) and Scotland (2210 cases and 9350 controls) the associations found in the Czech discovery set were not confirmed. However, expression analysis in human gut-related tissues and immune cells revealed that the NLRs associated with CRC risk or survival in the discovery set were expressed in primary human colon or rectum cells, CRC tissue and/or cell lines, providing preliminary evidence for a potential involvement of NLRs in general in CRC development and/or progression. Most interesting was the finding that the enigmatic development-related *NLRP5* (also known as *MATER*) was not expressed in normal colon tissue but in colon cancer tissue and cell lines. Future studies may show whether regulatory variants instead of coding variants might affect the expression of NLRs and contribute to CRC risk and survival.

## Introduction

Within the last few years it has become evident that the interplay between pattern recognition receptors (PRRs), such as Toll-like receptors (TLRs) or Nod-like receptors (NLRs), and the gut microbiota has a profound influence on the homeostasis of the immune system and therefore on many important aspects of human health [1–3]. If undisturbed and well-regulated, this symbiosis is beneficial for the human host. A disruption of the underlying regulatory pathways can, however, result in the development of local and chronic inflammation, inflammatory bowel disease (IBD) and/or colorectal cancer (CRC) [4, 5]. Gut homeostasis is maintained by a physical separation of the microbial community from the gut epithelia by the mucosa and a mucus layer. PRRs monitor the integrity of this barrier and the adjacent microbial community by detecting microbe-associated molecular patterns (MAMPs) as well as endogenous damage associated molecular patterns (DAMPs), and consequently controlling antimicrobial responses that contribute to an equilibrium between microbes and host [2, 4–6]. The activation of PRRs TLRs and/or NLRs by MAMPs or DAMPs results in the activation of multiple signaling pathways including nuclear factor-κB (NF-κB), mitogen-activated protein kinases (MAPKs), and the type I interferon (IFN) response, with subsequent induction of an inflammatory and anti-microbial response that includes secretory IgA, antimicrobial peptides, pyroptosis and autophagy [1, 2, 7]. Some NLRs, such as NLRP1, 3, 6, 12 and NLRC4, form so-called “inflammasome” complexes, comprising of the respective NLRs, the adaptor Apoptosis-associated speck-like protein containing a CARD (ASC) and pro-caspase-1. Inflammasome assembly initiates inflammatory and antimicrobial response via the autoproteolytic cleavage of caspase-1, catalysing the proteolytic conversion of pro-interleukin-1β (IL-1ß) and other IL-1 family members into biologically active cytokines which drive inflammation [2, 8, 9].

We recently reported an impact of TLR polymorphisms on CRC survival [10, 11]. Given the suggested concerted action of TLR and NLR signaling [1, 2], the connection between NLRs and CRC seemed of special interest. Provoking studies in mice and association studies in humans have suggested that NLR signaling is involved in inflammatory bowel disease, chronic inflammation and gastrointestinal cancers, including CRC [6, 11, 12]. Apart from various reports on mice, convincing data directly connecting NLRs and human CRC are available only for *NOD1*, *NOD2* and *NLPR3*, which were found to be associated with susceptibility, progression and treatment of sporadic CRC, colitis and/or colitis-associated CRC [13, 14]. In general, it is unclear whether and how other NLRs contribute to human CRC development or progression. Additionally, the functional importance of NLRs that have embryonic lethal phenotypes in mice–so-called “reproduction-related NLRs” like NLRP2, 5 and 13 –in human immunity and/or tumorigenesis remains an unresolved question [8].

In order to systematically investigate the influence of potentially functional coding polymorphisms in the NLR genes on sporadic CRC risk and survival, we conducted a case-control study with replications in 2 large genome-wide association studies (GWASs), covering the majority of known non-synonymous single nucleotide polymorphisms (nsSNPs) in 21 genes across different NLR signalling pathways. *In silico* analysis was done on selected nsSNPs in the NLR gene family. Furthermore, RNA expression of selected genes was measured to assess mRNA expression in immune cells, biopsies and/or CRC cell lines.

## Methods and material

### Ethical approval

The Czech study: Ethics Committee of the Institute of Experimental Medicine, Academy of Sciences of the Czech Republic, 26.3.2004; Ethics Committee of the Institute of Clinical and Experimental Medicine and Faculty Thomayer Hospital, Prague, Czech Republic, 29.4.2009; and Ethics Committee of the General University Hospital, Prague, Czech Republic, 4.4.2011.

For the work in Tübingen: All patients or healthy blood donors included in gene expression analyses for this study provided their written informed consent before study inclusion. Approval for use of their biomaterials was obtained by the local ethics committee at the University of Tübingen, in accordance with the principles laid down in the Declaration of Helsinki. Terminal ileum/ colon biopsies were obtained from patients undergoing routine colonoscopy at the University Hospital Tübingen, buffy coats obtained from blood donations of healthy donors were received from the Center for Clinical Transfusion Medicine (ZKT) at the University Hospital Tübingen and whole blood from voluntary healthy donors was obtained at the University of Tübingen, Department of Immunology.

The DACHS study was approved by the ethics committee of the Medical Faculty of the University of Heidelberg (no. 310/2001). The DACHS study is registered: StudyBox no. ST-066, DRKS no. DRKS00011793

The work in Scotland was approved by the UK National Health Service Research Ethics Committee (approval references 13/SS/0248; 11/SS/0109 and 01/0/05).

### SNP selection and in silico analysis of conservation and functional relevance

**Fig 1:**
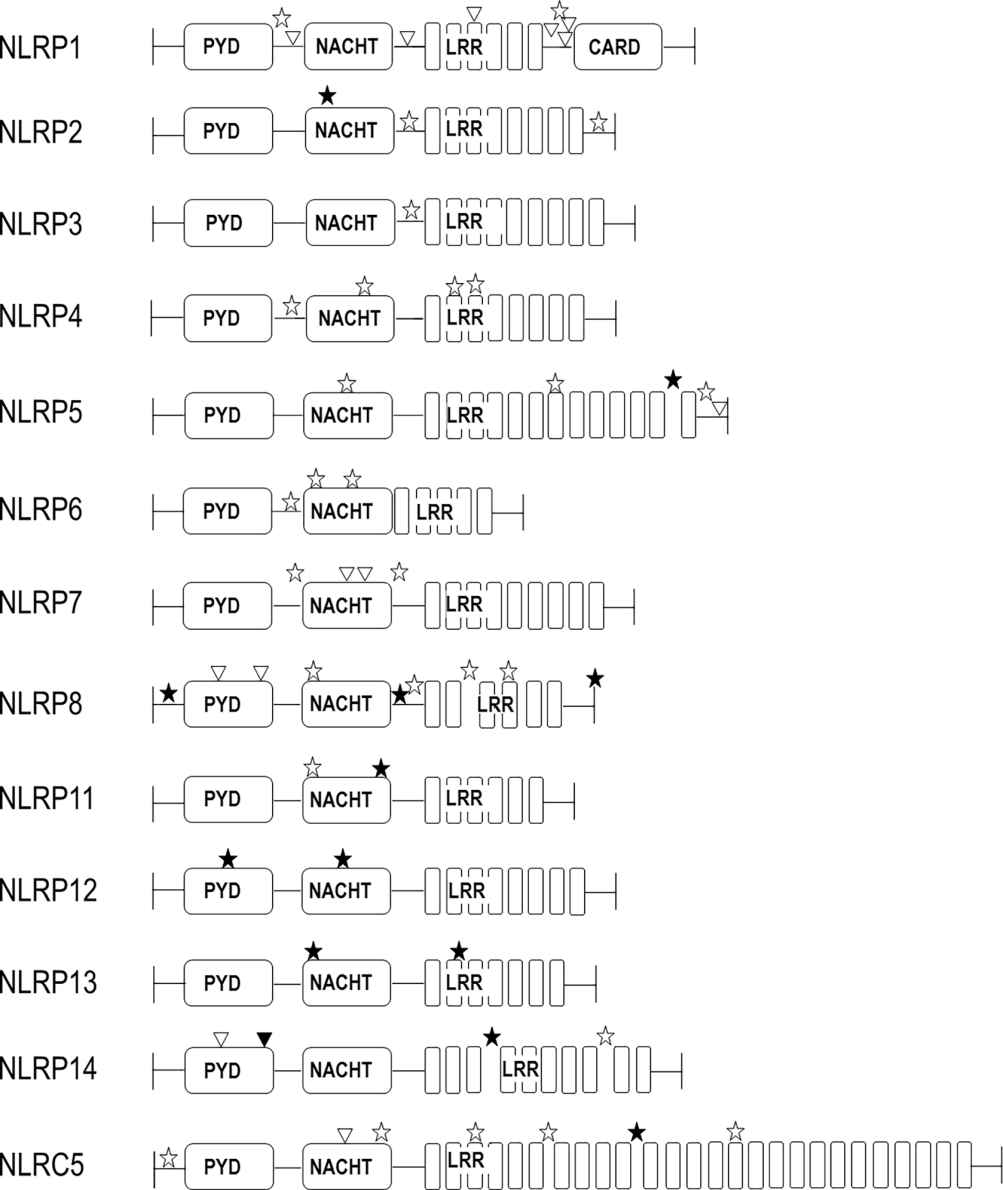
Protein structure of the candidate genes with genotyped SNPs (open and filled star symbols ☆/★), and all linked missense SNPs (triangle △; r^2^ ≥ 0.8). Filled star (★) and triangle (▲): SNPs predicted damaging (SIFT) or deleterious (Polyphen).

21 candidate NLR genes (NLRP1-14, NLRC4 and 5, NOD1 and 2, NAIP, RIPK2 and ASC [PYCARD]) were screened for non-synonymous variants. Thirteen of the 21 genes harboured validated missense variants with a minor allele frequency (MAF) > 0.01 in the CEU reference panels (Source: 1000Genomes, HapMap, dbSNP). Choosing only one SNP per linkage block (r^2^≥0.8), 41 SNPs were selected for genotyping (Table A in S2 File & Fig 1).

To gain additional insight into a possible functional relevance, all genotyped and linked SNPs were mapped to their location in the respective proteins. 19 of the 41 genotyped SNPs are located in defined NLR protein domains [3] (Fig 1 & Table A in S2 File): 11 in the NACHT domain, seven in the LRR domain and one in the PYD domain. The remaining genotyped SNPs mapped to linker regions. SIFT (sift.jcvi.org) and PolyPhen2 (genetics.bwh.harvard.edu/pph2) databases were used to assess possible effects of the SNPs on the protein. 23 of the genotyped SNPs are predicted to be deleterious or damaging, and/or to result in non-sense mediated decay or retained introns (Fig 1 & Table A in S2 File). Assessments of evolutionary conservation of the selected variants was performed by three software suites namely Genomic Evolutionary Rate Profiling (GERP [15]), PhastCons [16] and phylogenetic p-value (PhyloP [17]). The GERP score of >2.0 and the PhastCons score (values between 0–1) of >0.3 indicate a good level of conservation of the variants. Positive PhyloP scores (values between −14 and +6) are predicted to be conserved. Higher values of these tools reflect the probability that the nucleotide is located at a conserved position, based on the multiple alignment of genome sequences of 100 different vertebrates. Lower values of these tools reflect fast-evolving variant positions.

### Discovery set—Czech Republic

#### Study population

The study was carried out on a Czech CRC case-control population of patients (n = 1237; median age 63 years; 61.7% males) with colon or rectal malignancy—excluding hereditary-nonpolyposis colorectal cancer (HNPCC)—and healthy blood donors (n = 787; median age 47 years, 55.4% males, all cancer-free at the time of sampling), (Table B in S2 File.) [18]. For overall survival (OS) 477 incident CRC cases with information about age, sex, TNM staging, tumor grade, date of death or end of follow-up (August 31, 2011; median follow-up 58 months) were available. For event-free survival (EFS) in patients with non-metastatic disease at the time of diagnosis (n = 325), date of distant metastasis, tumor recurrence, death, or end of follow-up was used as the study end point (median follow-up 55 months).

#### SNP genotyping

TaqMan SNP Genotyping Assays (Applied Biosystems) or KASP genotyping assays (LGC Genomics) were used for the analysis of the SNPs. Case and control samples were amplified simultaneously in 384 well format (Hydrocycler 16 (LGC Genomics), using 3 ng whole genome amplified DNA from blood). Endpoint genotype detection was carried out on the ViiA 7 Real-Time PCR System (Applied Biosystems). Call rates for 40 out of 41 SNPs were 94–99%. Internal quality controls showed a concordance rate of ≥ 99%. Samples with < 50% call rate over all assays were excluded from the study.

### Replication sets—Germany and Scotland

For replication, all SNPs associated with CRC in the Czech population (p<0.05) were tested in two large European genome-wide association studies (GWASs) carried out in Germany („Darmkrebs: Chancen der Verhütung durch Screening Study”–DACHS) [19, 20] and in Scotland (Table B in S2 File) [21, 22].

#### Germany

The sample set used as the replication set is part of the still on-going DACHS project and comprised 1796 CRC patients (median age 69 years; 58.6% males) who received in-patient treatment due to a first diagnosis of CRC in 22 hospitals of the Rhine-Neckar-Odenwald region of Germany. The 1810 community-based controls were randomly selected from population registries matched for gender, 5-year age groups and county of residence (median age 70 years, 59.6% males, cancer-free at the time of sampling), (Table B in in S2 File) [19, 20]. Cases and controls genotyped in the present study were recruited between January 01, 2003 and December 31, 2007. For overall survival (OS) analysis, 1794 incident CRC cases with information about age, sex, tumor stage, and a median follow-up time of 48.4 months in men and 49.9 months in women were available [23].

Cases and controls were genotyped on the Illumina HumanCytoSNP or Illumina HumanOmniExpress platform [24]. Imputation was performed for autosomal SNPs to the CEU population in HapMap II release 24 using MACH (available at: www.sph.umich.edu/csg/abecasis/MACH/tour/) [24] with MAF (<0.01) and imputation accuracy (R2 < 0.3) excluded from the analysis [25].

#### Scotland

The Scottish study series comprised 2115 cases (median age 57 years, 57% males) from the Scottish colorectal cancer study (SOCCS) [21] and 95 cases (median age 67 years, 66% males) from Ninewells Hospital, Dundee and Perth Royal Infirmary collected between 1997 and 2000 [26]. SOCCS is a case-control study designed to identify genetic and environmental factors associated with non-hereditary CRC risk and survival outcome. Population controls with no personal history of cancer were ascertained from four cohorts including 8533 (42% males, mean age 55.4 yrs)—from Generation Scotland-Scottish Family Health Study [27, 28]; 513 (41% males, mean age 79 years) and 1004 (50.6% males, mean age 70 years) from the Lothian Birth Cohorts 1921 and 1936, respectively; and 262 Dundee controls (50% males) were recruited through the same General Practice surgeries as cases or from spouses/friends of cases [29]. The detailed information on genotyping cases and controls and data quality control is described elsewhere [22]. 2210 cases and 9350 controls were included in the final analysis. The survival analysis was performed in a subset of SOCCS study comprising 1402 patients (median follow up 107 months, recruited between 2001 and 2006) with colorectal adenocarcinoma confirmed by pathological assessment. Participants completed a detailed lifestyle questionnaire and a semi-quantitative food frequency and supplements questionnaire (http://www.foodfrequency.org). Genotyping was performed using the Infinium Human Exome BeadChip 12v1.0 or 12v1.1 (Illumina), with genotype calling using Illumina GenCall for HumanExome-12v1.0 and HumanExome-12v1.1 versions called separately. Generation Scotland controls and a subset of the cases from the SOCCS study were genotyped using OmniExpressExome BeadChip 8v1.1 or 8v1.2 (Illumina).

In accordance with the Declaration of Helsinki, all participants provided written informed consent. The studies were approved by the local ethics committees.

### Statistical analysis—Discovery set

Genotype frequencies in controls were tested for Hardy-Weinberg equilibrium (HWE; Pearson's goodness-of-fit χ^2^ test, deviation assumed at p < 0.001). *NLRP11* rs12461110 was excluded for violation of HWE.

#### Single variant associations with CRC risk, overall and event-free survival

Odds ratios (ORs) and 95% confidence intervals (CIs) for associations between genotypes and CRC risk were estimated by logistic regression (PROC LOGISTIC, SAS V9.3; SAS Institute, Cary, NC) and refer to the minor allele. P values were considered nominally significant at p≤0.05, with a study-wide significance level at p≤0.001 considering Bonferroni correction for multiple testing (0.05/39 = 0.0012). ORs were adjusted for age and sex. The estimated power was > 95% for OR ≥1.5 (MAF > 5%; p = 0.05; dominant model) [30].

Differences in OS and EFS between genotypes were estimated by hazard ratios (HRs) and 95% CIs using Cox regression (PROC PHREG, SAS V9.3) adjusting for age, sex, tumor grade and tumor stage. The estimated power was > 90% for HR ≥ 2.0 (MAF > 10%, p = 0.05). OS was calculated for all patients (OS_(pM0&1)_); for patients with non-metastatic disease at the time of diagnosis OS (OS_(pM0)_) and EFS (EFS_(pM0)_) were calculated. Kaplan-Meier plots were generated, estimating the differences between the survival functions by log-rank test (PROC LIFETEST, SAS V9.3).

#### Additive SNP associations with CRC risk and survival

Additive influence of the risk alleles (p≤0.05) on CRC risk and survival identified in the Czech population was estimated (risk: five SNPs, 0–10 risk alleles per individual; survival: eight SNPs, 0–16 risk alleles). For each SNP the allele associated with a higher OR or HR was designated the “risk allele”. Patients were grouped into equally sized groups of risk alleles (risk: 0-3/4-5/6-10; survival: 0-5/6-7/8-12) and ORs and HRs were calculated, adjusting for age and sex, HRs also for tumor grade and stage. Kaplan-Meier plots were generated for the additive survival model and the log-rank test was performed. The same analysis was conducted separately for the three *NLRP5* risk SNPs (p≤0.05) (0–6 risk alleles).

#### Statistical analysis—Replication sets

Data provided by the DACHS study consisted mostly of imputed genotypes, all in dosage format referring to the number of copies of minor allele. To permit direct comparison with the German data set genotype data from the Czech study was coded as 0, 1, or 2 copies of the minor allele. For these two data sets, association between SNPs and risk for CRC was obtained by applying logistic regression considering a log-additive genetic effect model (PROC LOGISTIC, SAS Version 9.2; SAS Institute). HRs (PROC PHREG, SAS version 9.2, SAS Institute) were calculated via Cox regression with a model that included the SNP coded as number of copies of the minor allele, age, sex and tumor stage for both sample sets.

ORs and 95% CIs for association between each of the genotypes and risk of CRC in Scotland were estimated using unconditional logistic regression adjusted for age and gender. HRs and corresponding 95%CI for overall survival analysis in Scotland was calculated for each of the genotyped SNPs and dominant model using Cox regression adjusted for age, gender and TNM stage. OS was calculated for all patients (OS_(pM0&1)_) and for patients with non-metastatic disease at the time of diagnosis OS (OS_(pM0)_). No event-free survival analysis was performed in the Scottish data. All analysis was performed in R v3.1.0 (R Development Core Team. R: A Language and Environment for Statistical Computing. Vienna: R Foundation for Statistical Computing, 2014).

### Gene expression

For expression analyses, patients providing biopsy material were recruited at the University Hospital Tübingen. Healthy blood donors were recruited at the Center for Clinical Transfusion Medicine (ZKT), University Hospital Tübingen and respective buffy coats obtained from blood donations. All patients/ healthy blood donors included in gene expression analyses for this study provided their written informed consent before study inclusion. Approval for use of their biomaterials was obtained by the local ethics committee at the University of Tübingen, in accordance with the principles laid down in the Declaration of Helsinki. Terminal ileum/ colon biopsies were obtained from patients undergoing routine colonoscopy at the University Hospital Tübingen, buffy coats obtained from blood donations of healthy donors were received from the Center for Clinical Transfusion Medicine (ZKT) at the University Hospital Tübingen and whole blood from voluntary healthy donors was obtained at the University of Tübingen, Department of Immunology.

#### Cell lines, primary human immune cells and biopsy material

HCT116, DLD-1 and Caco2 cells were grown and sourced as described [31], without re-authentication. Primary leukocytes were isolated from buffy coats (Tübingen University Hospital, Center for Clinical Transfusion Medicine (ZKT)) using Ficoll (GE Healthcare) density gradient purification and CD14+ monocytes were isolated using MACS (Miltenyi) magnetic beads to a purity of > 95% (anti-CD14-PE flow cytometry, BD). Subsequently, cells were differentiated into monocyte-derived dendritic cells (MoDC) or monocyte-derived macrophages (MoMacs) by culture in RPMI 1640 medium (Invitrogen) supplemented with 10% fetal calf serum (FCS) in the presence of 40 ng/ml IL-4 and 25 ng/ml GM-CSF (Peprotech) or with 25 ng/ml GM-CSF for 6 days, respectively. Neutrophils were isolated from the Ficoll pellet after NH_4_Cl lysis of erythrocytes. All cells were grown at 37°C and 5% CO_2_. Biopsies from the terminal ileum or colon (n = 12; median age 46; 56% males) were obtained during routine colonoscopy at the University Hospital Tübingen and stored in liquid nitrogen until analysis [13].

#### Gene expression analysis

Gene expression analysis was carried out using single-gene TaqMan® Gene Expression Assays (Applied Biosystems) for *NLRP2*, *NLRP3*, *NLRP5*, *NLRP6*, *NLRP13* and *NLRC5*. mRNA was isolated from whole blood or primary blood cells (two donors, #1 and #2, respectively) or THP-1, HCT116, DLD-1 or CaCo2 cell lines using an RNeasy Mini Kit (Qiagen) and commercially available RNA samples for human ovary, duodenum, ileum (sample #7), rectum and colon adenocarcinoma were used (Agilent). RNA from ileum or colon biopsies from six patients (samples #1–6) was isolated using TRIzol Reagent (Life Technologies) according to standard protocols and reverse transcribed into cDNA using oligo(dT)12 primer [13]. Following transcription to cDNA (High Capacity RNA-to-cDNA Kit; Life Technologies), expression was analysed using pre-validated TaqMan® Gene Expression Assays (Applied Biosystems). Data were normalized to *TBP* (TATA box binding protein). The samples were analysed in triplicate using the 7500fast Real-Time System (Applied Biosystems).

## Results

### NLR variants are associated with CRC risk and survival in the Czech sample set

Nominally significant associations with CRC risk were detected for six SNPs (Table 1; Table C in in S2 File). In an additive risk model combining those six variants, CRC risk increased significantly with increasing numbers of risk alleles, and a maximum for carriers of 6–10 risk alleles (OR 2.10, p = 0.0005; Table 1).

**Table 1:** CRC risk: genotype distribution of SNPs analyzed in the Czech case-control population for SNPs with p ≤ 0.05. Amino acid changes are given as <> with the amino acid position indicated. Data adjusted for age at diagnosis and sex. Nominal significance at p≤0.05; significance level corrected for multiple testing (39 genotyped SNPs) at p≤0.001.

Gene	Risk of CRC				
SNP	Genotype	Cases	Controls	OR (95%CI)	P Val
**NLRP2**	C/C	427	284	1	
**rs1043673**	A/C	574	355	1.08 (0.84–1.39)	0.56
**1052: A<>E**	A/A	203	108	1.41 (1.00–1.99)	**0.05**
	A/C + A/A	777	463	1.16 (0.91–1.47)	0.23
**NLRP3**	C/C	1114	700	1	
**rs35829419**	A/C	85	66	0.63 (0.41–0.97)	**0.04**
**705: Q<>K**	A/A	4	1	0.97 (0.09–10.11)	0.98
	C/A + A/A	89	67	0.64 (0.42–0.98)	**0.04**
**NLRP6**	G/G	924	629	1	
**rs6421985**	T/G	252	128	1.36 (1.01–1.83)	**0.04**
**163: L<>M**	T/T	-	-	-	-
	T/G + T/T	252	128	1.36 (1.01–1.83)	**0.04**
**NLRP8**	G/G	732	493	1	
**rs306457**	C/G	415	235	1.13 (0.88–1.45)	0.32
**1049: STOP<>Y**	C/C	63	26	2.01 (1.09–3.72)	**0.03**
	C/G + C/C	478	261	1.20 (0.95–1.53)	0.13
**NLRP11**	A/A	1070	662	1	** **
**rs299163**	A/C	134	91	0.94 (0.66–1.34)	0.74
** 188: A<>S**	C/C	7	12	0.21 (0.06–0.68)	**0.01**
** **	A/C + C/C	141	103	0.83 (0.59–1.17)	0.2954
**NLRP13**	C/C	419	297	1	
**rs303997**	C/T	564	334	1.37 (1.06–1.77)	**0.02**
**247: R<>Q**	T/T	212	125	1.54 (1.10–2.16)	**0.01**
	C/T + T/T	776	459	1.42 (1.11–1.80)	**0.005**
**No. of**	0–3	302 (27.83)	216 (32.34)	1	-
**risk alleles** ^**a**^	4–5	633 (58.34)	381 (57.04)	1.36 (1.04–1.79)	**0.03**
	6–10	150 (13.82)	71 (10.63)	2.10 (1.38–3.20)	**0.0005**

^a^ NLRP11 rs299163 was excluded from the “No. of risk alleles analysis” due to low MAF 0.05: only the rare homozygote genotype was associated with CRC risk, not contributing in risk in the Risk-SNP-Panel.

Eight SNPs were associated with altered OS and/or EFS (p≤0.05); Table 2; Table C in in S2 File). Strikingly, among them three unlinked SNPs in *NLRP5* (r^2^ < 0.5) were associated with decreased OS_(pM0)_ and EFS_(pM0)_. The additive survival model showed a nominally significantly decreased OS and EFS with an increasing number of risk alleles (Table 2). The maximum effect was detected for carriers of 8–12 risk alleles (HR_OS(pM0&1)_ 1.88, p = 0.003; HR_OS(pM0)_ 2.89 p = 0.0008 and HR_EFS(pM0)_ 3.02, p = 0.0003, respectively; Table 2).

**Table 2:** Overall survival pM0&1 and pM0, and event-free survival pM0: genotype distribution of SNPs analyzed in the Czech case-control population for SNPs with p ≤ 0.05. Amino acid changes are given as <> with the amino acid position indicated. Data adjusted for age at diagnosis and sex, tumor grade and tumor stage. Nominal significance at p ≤ 0.05; significance level corrected for multiple testing at p ≤ 0.001.

Gene		Overall Survival (pM = 0&1)				Overall Survival (pM = 0)				Event-free Survival (pM = 0)			
SNP	Genotype	Cases	Death (%)	HR (95%CI)	p-val	Cases	Deaths (%)	HR (95%CI)	p-val	Cases	Events (%)	HR (95%CI)	p-val
**NLRP1**	A/A	106	46 (43.40)	1		74	24 (32.43)	1		74	29 (39.19)	1	
**rs12150220**	A/T	177	86 (48.59)	1.14 (0.79–1.63)	0.49	130	43 (33.08)	1.10 (0.67–1.83)	0.70	130	50 (38.46)	0.95 (0.60–1.51)	0.81
** 155: H<>L**	T/T	78	42 (53.85)	1.57 (1.03–2.40)	**0.04**	59	25 (42.37)	1.52 (0.86–2.68)	0.15	59	27 (45.76)	1.31 (0.77–2.21)	0.32
** **	A/T+T/T	255	128 (50.20)	1.25 (0.89–1.76)	0.2	189	68 (35.98)	1.23 (0.77–1.97)	0.39	189	77 (40.74)	1.05 (0.68–1.62)	0.82
**NLRP2**	C/C	125	73 (58.40)	1		87	38 (43.68)	1		87	42 (48.28)	1	
**rs1043673**	A/C	181	71 (39.23)	0.64 (0.46–0.89)	**0.008**	136	38 (27.94)	0.59 (0.37–0.92)	**0.02**	136	45 (33.09)	0.61 (0.40–0.93)	**0.02**
** 1052: A<>E**	A/A	56	30 (53.57)	0.83 (0.54–1.28)	0.4	40	15 (37.50)	0.77 (0.42–1.42)	0.41	40	16 (40.00)	0.75 (0.42–1.35)	0.34
** **	A/C + A/A	237	101 (42.62)	0.69 (0.50–0.93)	**0.02**	176	53 (30.11)	0.63 (0.41–0.96)	**0.03**	87	42 (48.28)	0.64 (0.43–0.96)	**0.03**
**NLRP5**	G/G	197	88 (44.67)	1		143	42 (29.37)	1		143	48 (33.57)	1	
**rs10409555**	A/G	140	68 (48.57)	1.20 (0.87–1.64)	0.3	104	39 (37.50)	1.53 (0.98–2.38)	0.06	104	47 (45.19)	1.56 (1.04–2.35)	**0.03**
** 1181: V<>I**	A/A	25	18 (72.00)	1.58 (0.93–2.69)	0.09	17	10 (58.82)	3.04 (1.48–6.23)	**0.002**	17	10 (58.82)	2.36 (1.17–4.78)	**0.02**
** **	A/G + A/A	165	86 (52.12)	1.26 (0.93–1.70)	0.13	121	49 (40.50)	1.69 (1.11–2.58)	**0.02**	121	57 (47.11)	1.66 (1.12–2.45)	**0.01**
**NLRP5**	C/C	268	122 (45.52)	1		195	61 (31.28)	1		195	69 (35.38)	1	
**rs12462795**	C/G	90	47 (52.22)	1.28 (0.91–1.81)	0.15	68	28 (41.18)	1.71 (1.07–2.74)	**0.03**	68	34 (50.00)	1.80 (1.17–2.76)	**0.007**
** 1108: S<>C**	G/G	6	5 (83.33)	2.79 (1.13–6.90)	**0.03**	3	2 (66.67)	3.05 (0.73–12.74)	0.13	3	2 (66.67)	2.72 (0.66–11.29)	0.17
** **	C/G + G/G	96	52 (54.17)	1.36 (0.97–1.89)	0.07	71	30 (42.25)	1.77 (1.12–2.81)	**0.02**	71	36 (50.70)	1.84 (1.21–2.79)	**0.005**
**NLRP5**	T/T	253	118 (46.64)	1		184	57 (30.98)	1		184	64 (34.78)	1	
**rs16986899**	C/T	99	54 (54.55)	1.21 (0.87–1.67)	0.26	73	33 (45.21)	1.70 (1.10–2.64)	**0.02**	73	39 (53.42)	1.74 (1.17–2.61)	**0.007**
** 912: M<>T**	C/C	8	2 (25.00)	0.27 (0.07–1.13)	0.07	4	0 (0.00)	0.00 (0.00-.)	0.98	4	0 (0.00)	0.00 (0.00-.)	0.98
** **	C/T + C/C	107	56 (52.34)	1.08 (0.78–1.49)	0.64	77	33 (42.86)	1.58 (1.02–2.44)	**0.04**	77	39 (50.65)	1.61 (1.08–2.40)	**0.02**
**NLRP12**	C/C	225	112 (49.78)	1		161	60 (37.27)	1		161	71 (44.10)	1	
**rs34436714**	A/C	113	50 (44.25)	0.93 (0.66–1.30)	0.67	83	23 (27.71)	0.72 (0.45–1.17)	0.19	83	26 (31.33)	0.67 (0.43–1.06)	0.09
** 42: K<>N**	A/A	16	6 (37.50)	0.76 (0.33–1.73)	0.51	13	3 (23.08)	0.51 (0.16–1.65)	0.26	13	3 (23.08)	0.47 (0.15–1.49)	0.2
** **	A/C + A/A	129	56 (43.41)	0.91 (0.65–1.26)	0.55	96	26 (27.08)	0.69 (0.43–1.10)	0.12	96	29 (30.21)	0.64 (0.42–0.99)	**0.05**
**NLRC5**	C/C	192	101 (52.60)	1		133	53 (39.85)	1		133	61 (45.86)	1	
**rs289723**	A/C	149	65 (43.62)	0.87 (0.64–1.20)	0.4	113	33 (29.20)	0.72 (0.46–1.11)	0.14	113	37 (32.74)	0.61 (0.41–0.93)	**0.02**
** 1105: Q<>K**	A/A	27	11 (40.74)	0.99 (0.53–1.86)	0.98	23	8 (34.78)	1.13 (0.53–2.41)	0.75	23	10 (43.48)	1.04 (0.53–2.04)	0.92
** **	A/C + A/A	176	76 (43.18)	0.89 (0.66–1.20)	0.44	136	41 (30.15)	0.77 (0.51–1.17)	0.22	136	47 (34.56)	0.67 (0.46–0.99)	**0.04**
**NLRC5**	C/C	282	128 (45.39)	1		204	64 (31.37)	1		204	73 (35.78)	1	
**rs74439742**	C/T	75	43 (57.33)	1.42 (1.00–2.00)	**0.05**	56	26 (46.43)	1.67 (1.05–2.65)	**0.03**	56	31 (55.36)	1.63 (1.06–2.49)	**0.03**
** 191: P<>L**	T/T	10	7 (70.00)	2.39 (1.10–5.19)	**0.03**	7	4 (57.14)	3.07 (1.07–8.80)	**0.04**	7	4 (57.14)	2.71 (0.96–7.67)	0.06
** **	C/T + T/T	85	50 (58.82)	1.50 (1.08–2.09)	**0.02**	63	30 (47.62)	1.77 (1.13–2.75)	**0.01**	63	35 (55.56)	1.70 (1.13–2.57)	**0.01**
**No. of**	0–5	97	34 (35.05)	1	-	75	15 (20.00)	1	-	75	16 (21.33)	1	-
**risk alleles**	6–7	110	52 (47.27)	1.43 (0.92–2.21)	0.11	79	26 (32.91)	1.53 (0.81–2.90)	0.19	79	32 (40.51)	2.06 (1.12–3.77)	**0.02**
** **	8–12	108	62 (57.41)	1.88 (1.23–2.86)	**0.003**	75	33 (44.00)	2.89 (1.55–5.37)	**0.0008**	75	37(49.33)	3.02 (1.66–5.48)	**0.0003**

### GWAS data on the NLRP risk and survival SNPs—Replication sets

In order to validate the results from the Czech cohort, all SNPs included into the additive model for CRC risk (N = 5) and survival (N = 8) were analyzed in two large GWAS sample sets from Germany and Scotland. Complete data was available from the DACHS GWAS. The Scottish GWAS provided data on three CRC risk variants (rs12150220, rs306457 and rs303997) and seven survival variants (Table E in in S2 File). Scottish data was available for genotypes, DACHS data according to allelic probabilities. Despite the promising initial results, neither the associations for CRC risk nor the associations for CRC survival were replicated in the GWAS sets. We also tested the additive models in the DACHS population, but no association was evident (data not shown).

### Divergent expression patterns of NLRP2, 5, 6 and 13 in hematopoietic and non-hematopoietic cells

**Fig 2:**
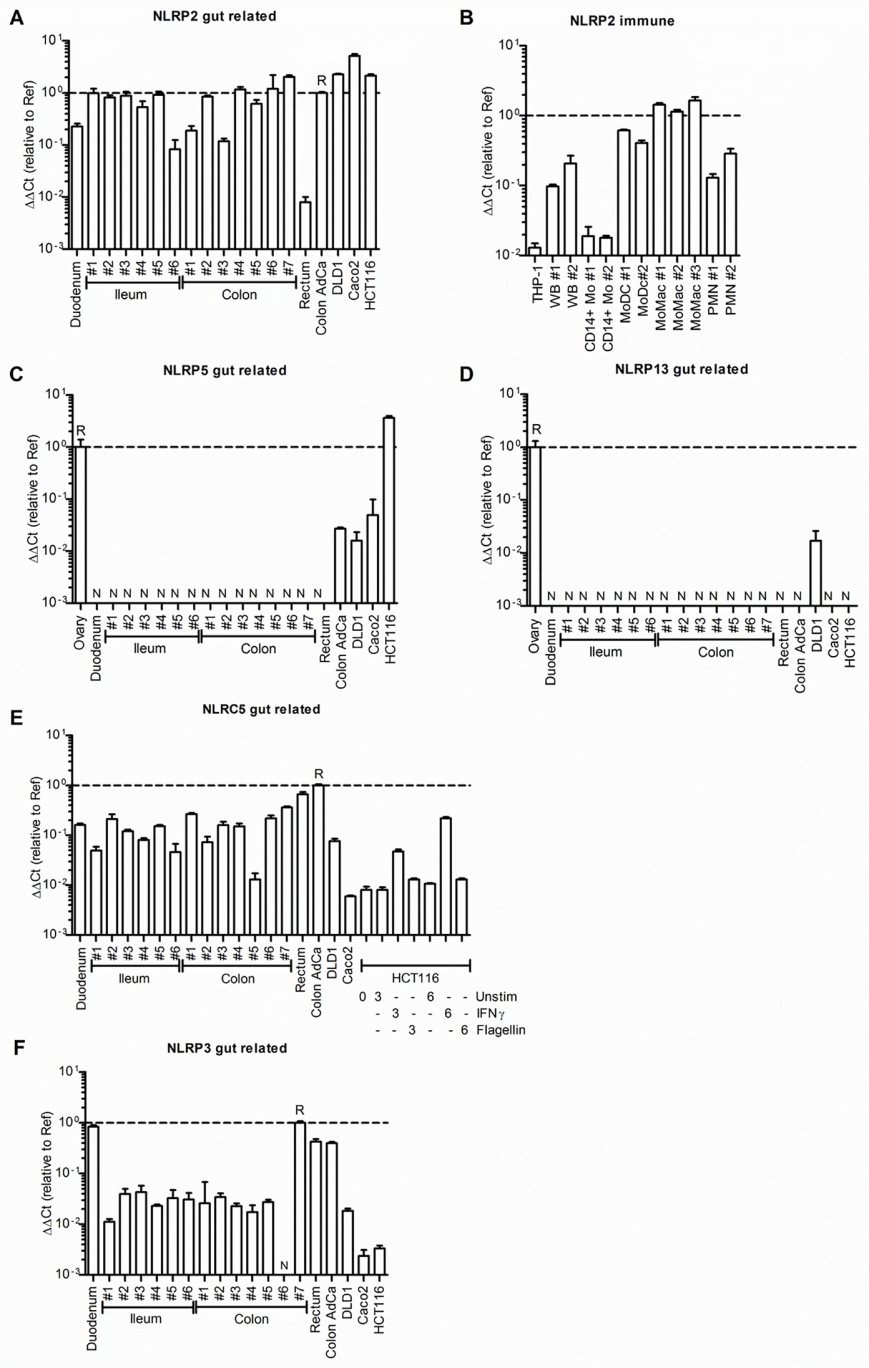
Expression of selected CRC-associated NLRs in immune cells, primary tissue samples or CRC cell lines. mRNA expression of NLRP2 (A,B), NLRP5 (C), NLRP13 (D), NLRC5 (E) and NLRP3 (F) was determined relative to the housekeeper TBP by performing triplicate (means +SD show) qPCR using TaqMan gene-specific primers and probes on the indicated samples (see Methods). In the case of (E) HCT cells were treated with 1000 U/ml IFNγ or 50 ng/ml S. typhimurium Flagellin for 3 or 6 hours as indicated. TBP-relative ΔCt values were normalized to a reference sample (labelled R, ΔΔCt method). N denotes samples in which no expression was detectable above Ct within 40 cycles.

To investigate whether the NLRs found to be associated with CRC in the Czech discovery set, were expressed in the gut or immune cells, mRNA levels were quantified for selected NLRs in primary tissue samples and cell lines. In gut-related tissues, CRC cell lines, whole blood and neutrophils (PMN), *NLRP2* showed moderate expression, with the lowest expression in rectum (Fig 2A and 2B). Although *NLRP2* showed low expression in CD14^+^ monocytes, this was increased up to 100-fold in monocyte-derived primary dendritic cells and macrophages (Fig 2B). *NLRP5* (also known as *MATER*) was not detectable in immune cells (not shown) and normal gut tissue but in ovary (Fig 2C), in keeping with its role in oogenesis [8]. However, we observed expression in a primary CRC sample and three CRC cell lines (Fig 2C) but not fibroblast or B lymphocyte cell lines (not shown), in agreement with mRNA expression data from the GENT database (Figure A in S1 File). Consistently with an earlier report [32], *NLRP6* (also known as *PYPAF5*) was highly expressed in neutrophils (PMN), low in monocytes and MoDC but not detectable in gut biopsies (not shown). *NLRP13* was below the level of detection in all analysed samples except for the positive control, ovary and the DLD1 CRC cell line (Fig 2D). *NLRC5* was expressed to varying degrees in healthy gut tissue and most highly in colon adenocarcinoma, and was inducible by IFNγ in HCT116 cells (Fig 2E). *NLRP3* was expressed at considerable levels only in duodenum, rectum and CRC samples, but not in normal ileum and colon biopsies (Fig 2F). While the role of *NLRP13* remains unclear, additional data on the reported occurrence of somatic mutations in these genes in CRC suggest that *NLRP2*, *NLRP3* and *NLRP6* may impact CRC development and survival via immune cells, whereas *NLRP5* might be relevant in gut tissues themselves, possibly experiencing a re-expression after malignant transformation.

## Discussion

In the discovery set from the Czech Republic, five of 39 successfully tested SNPs were associated with CRC risk, and eight with CRC survival. An additive effect on CRC risk and survival was detected, resulting in a 2-fold increased risk and a 3-fold worse survival for carriers of ≥6 and ≥8 risk alleles, respectively. Despite these promising results in the Czech population, these associations could not be confirmed in the two large German and Scottish GWAS data sets.

This was surprising taking into account the *in silico* predictions about the functionality of the SNPs and the results of the expression analysis which showed that the genes *NLRP2*, *NLRP3* and *NLRP6* may impact CRC development via immune cells. Accordingly, differential expression of these genes may cause alterations in pathways providing the emerging hallmarks of cancer, such as evading immune clearance and tumor-promoting inflammatory responses [33]. For *NLRC5*, whose expression was induced in HCT116 cells by IFNγ (Fig 2E), one plausible functional outcome may be the modulation of MHC class I expression [34]. The latter strongly correlates with CRC survival due to its effect on CD8 cytotoxic T cell and natural killer cell immuno-surveillance [35]. According to murine data, the *NLRP12* may also affect T cell function in the context of human CRC [36]. Most intriguingly, expression of development-related *NLRP5* was undetectable in normal gut-related tissues but was up-regulated in malignant gut tissue and colon cancer cell lines (*cf*. Fig 2C and Figure A in S1 File.), suggesting for the first time a potential novel role beyond developmental control for this enigmatic NLR in humans [8]. During oogenesis, murine Nlrp5 appears to influence mitochondrial localization and activity, ATP content and Ca^2+^ homeostasis–processes which all have been linked to NLRP3 inflammasome activation and thus inflammation in differentiated cells. NLRP5 may thus act in concert with NLRP3, which is known to be associated with human CRC [14], a speculation warranting further investigation. The concerted association of genes of the NLR family may directly link environmental risk factors, intestinal inflammation, the microbiota and well-described cancer pathways involved in CRC development, such as the MAPK pathway and the NF-κB pathway.

One might argue that the failure to replicate the association results in the Czech discovery set might be due to differences in the clinical composition between the case-control populations of the discovery set and the replication sets (Table B in S2 File). However, data was adjusted for all significantly different parameters except tumor location (colon or rectum; not possible due to incomplete data) suggesting that the detected associations in the discovery set were false positive results. In the light of the supporting gene expression data it is possible that the coding variants analyzed in this study do actually not have an effect on the functionality of the receptor proteins. This assumption is supported by the fact that the majority of variants are not located in evolutionary conserved regions of the genes which allow for natural variability. Further, it is also possible that undetected environmental factors might have biased the results. Especially for immune related genetic variants, interactions with environmental factors or treatment might play a major role enhancing or even enabling effects of SNPs on CRC risk or survival. Based on the interesting expression results, future studies of these genes and their encoded receptors, including the analysis of regulatory genetic variants affecting the gene expression as well as the analysis of the patient specific tumor microenvironment and tumor infiltrating immune cells and immune constitution, may contribute to uncover the still poorly understood role of NLRs within the intestinal immune system, as well as, in CRC development and survival [37]. The integration of different exogenous, endogenous, tumour and immune factors, potentially including the variants in NLR genes studied here, holds promise for future approaches in precision medicine [37].

## Supporting information

S1 File (Figure A) mRNA expression of NLRP5 (MATER) in CRC.
(DOCX)

S2 File (Table A) Complete list of genotyped SNPs in candidate genes, with information about all linked missense SNPs (r^2^ ≥ 0.8) and the location in protein domains. NMD: nonsense mediated decay; * Variant Effect Predictor by Ensembl http://www.ensembl.org/Homo_sapiens/Tools/VEP (Table B) Population Description. a Z statistics: Wilcoxon Rank-Sum-Test; b Chi-square; event = recurrence, metastasis, death. (Table C) Genotype distribution of all analysed SNPs in the Czech case-control population: Risk and Survival analysis. CRC Risk: Data adjusted for age of diagnosis and sex. Overall Survival and Event free Survival: Data adjusted for age of diagnosis and sex, grade and stage. Nominal significance at p ≤ 0.05; significance level corrected for multiple testing at p ≤ 0.001. (Table D) mRNA Expression for the most promising candidate genes: Study data and reported somatic mutations for CRC-associated NLRs. (Table E) CRC risk and Overall survival pM0: Comparison of the SNPs with p ≤ 0.05 in the Czech discovery set with GWAS results from the Scottish and DACHS replication sets. Amino acid changes are given as &lt;&gt; with the amino acid position indicated. Nominal significance at p ≤ 0.05.
(DOCX)

## Abbreviations

ASC: Apoptosis-associated speck-like protein containing a CARD
CEU: Utah residents with Northern and Western European ancestry from the CEPH collection
CI: confidence interval
CRC: colorectal cancer
EFS: event-free survival
HNPCC: hereditary-nonpolyposis colorectal cancer
HR: hazard ratio
HWE: Hardy-Weinberg equilibrium
MAF: minor allele frequency
MoDC: monocyte-derived dendritic cells
MoMacs: monocyte-derived macrophages
NLRs: Nod-like receptors
nsSNPs: non-synonymous single nucleotide polymorphisms
OR: Odds ratio
OS: overall survival
pM 0: no metastasis upon diagnosis
pM1: metastasis upon diagnosis
PRRs: pattern recognition receptors
SNPs: single nucleotide polymorphisms
TLRs: Toll-like receptors
TNM: UICC TNM staging: size or direct extent of the primary tumor (T); degree of spread to regional lymph nodes (N); presence of metastasis (M)
