# Supplementary material for: Coding variants in NOD-like receptors: An association study on risk and survival of colorectal cancer
Source: PLoS One. 2018 Jun 21;13(6):e0199350. doi: 10.1371/journal.pone.0199350 (PMC6013205; doi:10.1371/journal.pone.0199350)
Supplement: S1 File — (Figure A) mRNA expression of NLRP5 (MATER) in CRC. (DOCX) [file pone.0199350.s001.docx]

**S1 Supplementary Figure**

**Coding variants in NOD-like receptors are associated with risk and survival of colorectal cancer**

**Content**

[**Figure A: mRNA expression of NLRP5 (MATER) in CRC.** 1](#_Toc516497176)


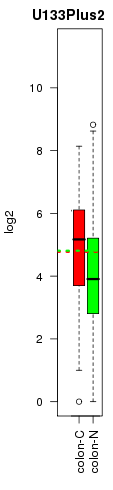


**Figure A: mRNA expression of NLRP5 (MATER) in CRC.**

Data were extracted for NLRP5 from the GENT database ([1](#_ENREF_1)) and extracting the data for CRC (C) and normal tissue (N).

**References:**

1. Shin G, Kang TW, Yang S, Baek SJ, Jeong YS, Kim SY. GENT: gene expression database of normal and tumor tissues. Cancer Inform. 2011;10:149-57.
