# Supplementary material for: Coding variants in NOD-like receptors: An association study on risk and survival of colorectal cancer
Source: PLoS One. 2018 Jun 21;13(6):e0199350. doi: 10.1371/journal.pone.0199350 (PMC6013205; doi:10.1371/journal.pone.0199350)
Supplement: S2 File — (Table A) Complete list of genotyped SNPs in candidate genes, with information about all linked missense SNPs (r2 ≥ 0.8) and the location in protein domains. NMD: nonsense mediated decay; * Variant Effect Predictor by Ensembl http://www.ensembl.org/Homo_sapiens/Tools/VEP (Table B) Population Description. a Z statistics: Wilcoxon Rank-Sum-Test; b Chi-square; event = recurrence, metastasis, death. (Table C) Genotype distribution of all analysed SNPs in the Czech case-control population: Risk and Survival analysis. CRC Risk: Data adjusted for age of diagnosis and sex. Overall Survival and Event free Survival: Data adjusted for age of diagnosis and sex, grade and stage. Nominal significance at p ≤ 0.05; significance level corrected for multiple testing at p ≤ 0.001. (Table D) mRNA Expression for the most promising candidate genes: Study data and reported somatic mutations for CRC-associated NLRs. (Table E) CRC risk and Overall survival pM0: Comparison of the SNPs with p ≤ 0.05 in the Czech discovery set with GWAS results from the Scottish and DACHS replication sets. Amino acid changes are given as <> with the amino acid position indicated. Nominal significance at p ≤ 0.05. (DOCX) [file pone.0199350.s002.docx]

**S2 Supplementary Tables**

**Coding variants in NOD-like receptors are associated with risk and survival of colorectal cancer**

**Content**

[**Table A: Complete list of genotyped SNPs in candidate genes, with information about all linked missense SNPs (r² ≥ 0.8) and the location in protein domains.** NMD: nonsense mediated decay; * Variant Effect Predictor by Ensembl http://www.ensembl.org/Homo_sapiens/Tools/VEP](#_Toc516497056)

[**Table B: Population Description**. ^a^ Z statistics: Wilcoxon Rank-Sum-Test; ^b^ Chi-square; event = recurrence, metastasis, death.](#_Toc516497057)

[**Table C: Genotype distribution of all analysed SNPs in the Czech case-control population: Risk and Survival analysis.** CRC Risk: Data adjusted for age of diagnosis and sex. Overall Survival and Event free Survival: Data adjusted for age of diagnosis and sex, grade and stage. Nominal significance at p ≤ 0.05; significance level corrected for multiple testing at p ≤ 0.001.](#_Toc516497058)

[**Table D: mRNA Expression for the most promising candidate genes:** Study data and reported somatic mutations for CRC-associated NLRs.](#_Toc516497059)

[**Table E: CRC risk and Overall survival pM0:** Comparison of the SNPs with p ≤ 0.05 in the Czech discovery set with GWAS results from the Scottish and DACHS replication sets. Amino acid changes are given as <> with the amino acid position indicated. Nominal significance at p ≤ 0.05.](#_Toc516497060)

**Table A: Complete list of genotyped SNPs in candidate genes, with information about all linked missense SNPs (r² ≥ 0.8) and the location in protein domains.** NMD: nonsense mediated decay; * Variant Effect Predictor by Ensembl http://www.ensembl.org/Homo_sapiens/Tools/VEP

| **Gene** | **SNP** | **Alleles** | **Position (GRCh37)** | **Amino Acid  Change** | **Function** | **Domain** | **MAF  (CEU)** | **linked nsSNPs** | **Effect on the protein  (transcript dependent)*** | **GERP++** | **phyloP 100way  vertebrate** | **phastCons 100way vertebrate** |
| --- | --- | --- | --- | --- | --- | --- | --- | --- | --- | --- | --- | --- |
| NLRP1 | rs12150220 | AT | 17:5485367 | L155H | missense |  | 0.46 |  |  | -1,07 | -1,192 | 0 |
| NLRP1 | rs35596958 | TC | 17:5433966 | M1123V | missense |  | 0.06 | r² > 0.95 rs2137722, rs11653832, 34733791, rs11657747 (LRR), rs52795654, rs11651595 | NMD | 2,58 | 1,165 | 0,223 |
| NLRP2 | rs1043673 | CA | 19:55512232 | A1052E | missense |  | 0.38 |  |  | -0,369 | -0,986 | 0 |
| NLRP2 | rs17699678 | CT | 19:55493728 | T221M | missense | NACHT | 0.15 |  | deleterious (SIFT), damaging (PolyPhen), NMD | 0,562 | 0,749 | 0,002 |
| NLRP2 | rs34804158 | AG | 19:55494651 | T529A | missense |  | 0.28 |  |  | -3,59 | -2,265 | 0 |
| NLRP3 | rs35829419 | CA | 01:247588858 | Q705K | missense |  | 0.06 |  | processed transcript | -0,977 | -0,197 | 0 |
| NLRP4 | rs12462372 | GA | 19:56373462 | R708H | missense | LRR | 0.07 |  |  | -6,52 | -2,22 | 0 |
| NLRP4 | rs17857373 | GC | 19:56369908 | E383D | missense | NACHT | 0.07 |  |  | -8,19 | -2,839 | 0 |
| NLRP4 | rs302453 | AT | 19:56390237 | Q925L | missense | LRR | 0.18 |  |  | -7,21 | -0,565 | 0 |
| NLRP4 | rs441827 | CT | 19:56369189 | A144T | missense |  | 0.4 |  |  | -6,89 | -0,167 | 0 |
| NLRP5 | rs10409555 | GA | 19:56572832 | V1181I | missense |  | 0.24 |  |  | -6,66 | -1,55 | 0 |
| NLRP5 | rs12462795 | CG | 19:56569629 | S1108C | missense |  | 0.15 | r² > 0.95 rs36118060 | deleterious (SIFT), damaging (PolyPhen) | 2,32 | 0,188 | 0 |
| NLRP5 | rs16986899 | TC | 19:56549510 | M912T | missense | LRR | 0.13 |  |  | -3,09 | -0,908 | 0 |
| NLRP5 | rs471979 | GC | 19:56538976 | M459I | missense | NACHT | 0.12 |  |  | -0,707 | -0,141 | 0 |
| NLRP6 | rs56159585 | TA | 11:280816 | Y361F | missense | NACHT | 0.14 |  | retained intron | 3,52 | 0,176 | 0,16 |
| NLRP6 | rs6421985 | GT | 11:280221 | M163L | missense |  | 0.05 |  | retained intron, regulatory feature | 2,78 | 0,249 | 0,002 |
| NLRP6 | rs77447196 | CG | 11:280464 | P244A | missense | NACHT | 0.11 |  | retained intron | -0,0364 | -2,845 | 0 |
| NLRP7 | rs61747414 | CT | 19:55450746 | A481T | missense |  | 0.14 |  |  | -3,83 | -1,466 | 0 |
| NLRP7 | rs7359934 | GA | 19:55447501 |  | intronic |  | 0.26 | r² > 0.95 rs10418277 (NACHT), rs775882 (NACHT) | NMD | -0,98 | -0,308 | 0 |
| NLRP8 | rs306457 | GC | 19:56499279 | STOP1049Y | STOP loss |  | 0.3 |  | Stop loss | 0,372 | -1,258 | 0 |
| NLRP8 | rs306481 | GA | 19:56487603 | K937R | missense | LRR | 0.47 |  |  | -5,02 | -1,002 | 0 |
| NLRP8 | rs306496 | AG | 19:56477710 | V782A | missense |  | 0.51 |  |  | 1,82 | 0,031 | 0 |
| NLRP8 | rs306507 | TC | 19:56459342 | P25L | missense |  | 0.42 | r² > 0.95 rs306506 (PYD) | deleterious (SIFT) | 0,802 | -0,435 | 0 |
| NLRP8 | rs41481648 | GA | 19:56467375 | R651W | missense |  | 0.06 |  | damaging (PolyPhen) | -3,03 | -2,175 | 0 |
| NLRP8 | rs61740015 | AG | 19:56485077 | Q865R | missense |  | 0.07 |  |  | -0,828 | -0,934 | 0 |
| NLRP8 | rs7259764 | AG | 19:56466227 | Q268R | missense | NACHT | 0.06 | r² > 0.85 rs61195059 (PYD) | -2,85 | -1,701 | 0 |  |
| NLRP11 | rs12461110 | GA | 19:56320663 | P438L | missense | NACHT | 0.33 |  | damaging (PolyPhen), NMD | -4,4 | -1,208 | 0 |
| NLRP11 | rs299163 | AC | 19:56321414 | A188S | missense | NACHT | 0.08 |  | regulatory feature | 0,306 | 1,235 | 0,001 |
| *NLRP12* | *rs12610233* | *AC* |  |  |  |  |  | *Failed to analyse* | *retained intron, regulatory feature* | *0,17* | *-0,2* | *0,023* |
| NLRP12 | rs34436714 | CA | 19:54327313 | G39V | missense | PYD | 0.19 |  | deleterious (SIFT), damaging (PolyPhen), regulatory feature | 4,48 | 1,527 | 0,016 |
| NLRP12 | rs34971363 | GC | 19:54313707 | F402L | missense | NACHT | 0.08 |  | deleterious (SIFT), damaging (PolyPhen), retained intron | -6,74 | -3,392 | 0 |
| NLRP13 | rs17711239 | TC | 19:55907897 | N781S | missense | LRR | 0.11 |  | damaging (PolyPhen) | 1,15 | 0,006 | 0 |
| NLRP13 | rs303997 | CT | 19:55913077 | Q247R | missense | NACHT | 0.35 |  | deleterious (SIFT) | 2,81 | 4,539 | 0,987 |
| NLRP14 | rs10839708 | AG | 11:7057807 | E808K | missense |  | 0.41 | r² > 0.80 rs1156526 (PYD) | damaging (PolyPhen) | 3,98 | 3,225 | 1 |
| NLRP14 | rs17280682 | CT | 11:7070338 | L1010F | missense |  | 0.23 | r² > 0.95 rs61063081 (PYD) | 4,13 | 1,99 | 0,891 |  |
| NLRC5 | rs289723 | CA | 16:57046616 | Q1105K | missense |  | 0.14 |  | NMD, retained intron | 1,52 | 0,884 | 0,001 |
| NLRC5 | rs3751710 | GA | 16:57061863 | L1386F | missense |  | 0.24 |  | deleterious (SIFT), retained intron, regulatory feature | 1,65 | 0,306 | 0 |
| NLRC5 | rs7185320 | AG | 16:57067461 | Q1466R | missense | LRR | 0.18 | r² > 0.95 rs7190199 | NMD | -7,14 | -1,451 | 0,119 |
| NLRC5 | rs74439742 | CT | 16:57025515 | P191L | missense |  | 0.07 |  | NMD, retained intron | 0,21 | -0,005 | 0 |
| NLRC5 | rs28438857 | TC | 16:57026441 | C500R | missense | NACHT | 0.11 | r² > 0.90 rs9938543 (NACHT) | NMD, retained intron | -5,88 | 0,375 | 0,014 |
| NLRC5 | rs78176773 | CT | 16:57037201 | S906R | missense | LRR | 0.31 |  | NMD, retained intron, regulatory feature | NA | NA | NA |

**Table B: Population Description**. ^a^ Z statistics: Wilcoxon Rank-Sum-Test; ^b^ Chi-square; event = recurrence, metastasis, death.

| **CRC Sample Sets** | | **Czech Sample Set** | | **German Sample Set** | | **Scottish Sample Set** | |
| --- | --- | --- | --- | --- | --- | --- | --- |
| **Risk Analysis** | | **Cases** | **Controls** | **Cases** | **Controls** | **Cases** | **Controls** |
| **Number of patients** | | 1237 | 787 | 1796 | 1810 | 2210 | 9350 |
| **Age at diagnosis** | Mean [range] | 62.04 [24 - 89] | 46.52 [18 - 94] | 68.21 [33 - 94] | 68.69 [34 - 98] | 56.76 [17-89] | 58.13 [18 -99] |
|  | Median | 63 | 47 | 69 | 70 | 57.46 | 59 |
| **Sex** | Male | 763 (61.68%) | 436 (55.40%) | 1052 (58.57%) | 1078 (59.56%) | 1269 (57.4%) | 3983 (42.6%) |
|  | Female | 474 (38.32%) | 351 (44.60%) | 744 (41.43%) | 732 (40.44%) | 941 (42.6%) | 5367 (57.4%) |
| **Tumour location** | Colon | 715 (57.80%) |  | 1097 (61.0%) |  | 1172 (53.03%) | |
|  | Rectum | 346 (27.97%) |  | 701 (39.0%) |  | 947 (42.85%) |  |
|  | Missing | 176 (14.23%) |  | - |  | 91 (4.12%) |  |
| **Survival Analysis** | | **pM0&1** |  | **pM0&1** |  | **pM0&1** |  |
| **Number of patients at risk** | | 477 |  | 1794 |  | 1402 |  |
| **Age at diagnosis** | Mean [range] | 63.42 [27 - 89] |  | 68.23 [33 - 94] | | - |  |
| **Grade** | 1 or 2 | 307 (64.36%) |  | 1137 (63.38%) | | - |  |
|  | 3 or 4 | 101 (21.17%) |  | 436 (24.30%) |  | - |  |
|  | Missing | 69 (17.47%) |  | 221 (12.32%) |  | - |  |
| **Stage** | I | 55 (11.53%) |  | 396 (22.07%) |  | 373 (16.88%) |  |
|  | II | 127 (26.62%) |  | 518 (28.87%) |  | 645 (29.29%) |  |
|  | III | 119 (24.95%) |  | 564 (31.44%) |  | 670 (30.32%) |  |
|  | IV | 126 (26.42%) |  | 268 (14.94%) |  | 335 (15.16%) |  |
| T | 0 | 87 (18.24%) |  | - |  | - |  |
|  | 1 | 346 (72.54%) |  | - |  | - |  |
|  | Missing | 44 (9.22%) |  | - |  | - |  |
| N | 0 | 215 (45.07%) |  | - |  | - |  |
|  | 1 | 187 (39.20%) |  | - |  | - |  |
|  | Missing | 75 (15.72%) |  | - |  | - |  |
| M | 0 | 319 (66.88%) |  | - |  | - |  |
|  | 1 | 126 (26.42%) |  | - |  | - |  |
|  | Missing | 32 (6.71%) |  | - |  | - |  |

**Table C: Genotype distribution of all analysed SNPs in the Czech case-control population: Risk and Survival analysis.** CRC Risk: Data adjusted for age of diagnosis and sex. Overall Survival and Event free Survival: Data adjusted for age of diagnosis and sex, grade and stage. Nominal significance at p ≤ 0.05; significance level corrected for multiple testing at p ≤ 0.001.

| **Gene** |  | **Risk of CRC** | | | | **Overall Survival (pM=0&1)** | | | | **Overall Survival (pM=0)** | | | | **Event free Survival (pM=0)** | | | |
| --- | --- | --- | --- | --- | --- | --- | --- | --- | --- | --- | --- | --- | --- | --- | --- | --- | --- |
| **SNP** | **Genotype** | **Cases** | **Controls** | **OR (95%CI)** | **P Val** | **Cases** | **Death (%)** | **HR (95%CI)** | **p-val** | **Cases** | **Deaths (%)** | **HR (95%CI)** | **p-val** | **Cases** | **Events (%)** | **HR (95%CI)** | **p-val** |
| **NLRP1** | A/A | 352 | 213 | 1 |  | 106 | 46 (43.40) | 1 |  | 74 | 24 (32.43) | 1 |  | 74 | 29 (39.19) | 1 |  |
| **rs12150220** | A/T | 599 | 366 | 1.11 (0.85-1.46) | 0.4355 | 177 | 86 (48.59) | 1.14 (0.79-1.63) | 0.49 | 130 | 43 (33.08) | 1.10 (0.67-1.83) | 0.7026 | 130 | 50 (38.46) | 0.95 (0.60-1.51) | 0.8187 |
|  | T/T | 242 | 161 | 1.02 (0.73-1.41) | 0.9261 | 78 | 42 (53.85) | 1.57 (1.03-2.40) | **0.0355** | 59 | 25 (42.37) | 1.52 (0.86-2.68) | 0.1452 | 59 | 27 (45.76) | 1.31 (0.77-2.21) | 0.3206 |
|  | A/T+T/T | 841 | 527 | 1.08 (0.84-1.40) | 0.5397 | 255 | 128 (50.20) | 1.25 (0.89-1.76) | 0.1969 | 189 | 68 (35.98) | 1.23 (0.77-1.97) | 0.3884 | 189 | 77 (40.74) | 1.05 (0.68-1.62) | 0.8211 |
| **NLRP1** | T/T | 1060 | 684 | 1 |  | 317 | 148 (46.69) | 1 |  | 233 | 78 (33.48) | 1 |  | 233 | 91 (39.06) | 1 |  |
| **rs35596958** | C/T | 150 | 92 | 0.89 (0.62-1.27) | 0.5112 | 46 | 28 (60.87) | 1.30 (0.86-1.96) | 0.2106 | 30 | 14 (46.67) | 1.58 (0.89-2.81) | 0.1193 | 30 | 14 (46.67) | 1.32 (0.75-2.34) | 0.3342 |
|  | C/C | 2 | 1 | 1.72 (0.07-44.91) | 0.7441 | 1 | 1 (100.00) | 10.83 (1.45-80.83) | **0.0202** | 1 | 1 (100.00) | 12.34 (1.58-96.24) | **0.0165** | 1 | 1 (100.00) | 6.01 (0.80-45.01) | 0.0806 |
|  | C/T+C/C | 152 | 93 | 0.89 (0.63-1.28) | 0.5355 | 47 | 29 (61.70) | 1.34 (0.90-2.01) | 0.1533 | 31 | 15 (48.39) | 1.68 (0.96-2.95) | 0.0679 | 31 | 15 (48.39) | 1.40 (0.81-2.43) | 0.2325 |
| **NLRP2** | C/C | 427 | 284 | 1 |  | 125 | 73 (58.40) | 1 |  | 87 | 38 (43.68) | 1 |  | 87 | 42 (48.28) | 1 |  |
| **rs1043673** | A/C | 574 | 355 | 1.08 (0.84-1.39) | 0.5566 | 181 | 71 (39.23) | 0.64 (0.46-0.89) | **0.0078** | 136 | 38 (27.94) | 0.59 (0.37-0.92) | **0.0213** | 136 | 45 (33.09) | 0.61 (0.40-0.93) | **0.0228** |
|  | A/A | 203 | 108 | 1.41 (1.00-1.99) | **0.05** | 56 | 30 (53.57) | 0.83 (0.54-1.28) | 0.3981 | 40 | 15 (37.50) | 0.77 (0.42-1.42) | 0.4114 | 40 | 16 (40.00) | 0.75 (0.42-1.35) | 0.3416 |
|  | A/C + A/A | 777 | 463 | 1.16 (0.91-1.47) | 0.2312 | 237 | 101 (42.62) | 0.69 (0.50-0.93) | **0.0151** | 176 | 53 (30.11) | 0.63 (0.41-0.96) | **0.0313** | 87 | 42 (48.28) | 0.64 (0.43-0.96) | **0.0288** |
| **NLRP2** | C/C | 1007 | 583 | 1 |  | 314 | 151 (48.09) | 1 |  | 231 | 80 (34.63) | 1 |  | 231 | 93 (40.26) | 1 |  |
| **rs17699678** | C/T | 193 | 153 | 0.76 (0.56-1.02) | 0.067 | 46 | 22 (47.83) | 0.93 (0.59-1.45) | 0.7366 | 31 | 11 (35.48) | 1.21 (0.64-2.30) | 0.551 | 31 | 11 (35.48) | 0.88 (0.47-1.66) | 0.7002 |
|  | T/T | 11 | 8 | 0.53 (0.16-1.73) | 0.2921 | 4 | 3 (75.00) | 1.21 (0.38-3.86) | 0.7486 | 2 | 1 (50.00) | 0.67 (0.09-5.15) | 0.7045 | 2 | 1 (50.00) | 0.65 (0.09-4.84) | 0.6726 |
|  | C/T + T/T | 204 | 161 | 0.74 (0.55-1.00) | 0.0566 | 50 | 25 (50.00) | 0.95 (0.62-1.46) | 0.8235 | 33 | 12 (36.36) | 1.14 (0.62-2.11) | 0.6711 | 33 | 12 (36.36) | 0.86 (0.47-1.58) | 0.624 |
| **NLRP2** | A/A | 622 | 407 | 1 |  | 187 | 88 (47.06) | 1 |  | 140 | 47 (33.57) | 1 |  | 140 | 58 (41.43) | 1 |  |
| **rs34804158** | G/A | 401 | 245 | 1.06 (0.82-1.37) | 0.6441 | 121 | 56 (46.28) | 1.08 (0.77-1.51) | 0.6712 | 90 | 32 (35.56) | 0.95 (0.61-1.49) | 0.822 | 90 | 34 (37.78) | 0.90 (0.59-1.37) | 0.6199 |
|  | G/G | 108 | 66 | 0.92 (0.61-1.37) | 0.6757 | 35 | 22 (62.86) | 0.96 (0.60-1.55) | 0.8815 | 21 | 9 (42.86) | 1.03 (0.50-2.12) | 0.9428 | 21 | 9 (42.86) | 0.95 (0.47-1.95) | 0.8996 |
|  | G/A + G/G | 509 | 311 | 1.03 (0.81-1.30) | 0.822 | 156 | 78 (50.00) | 1.04 (0.77-1.42) | 0.7925 | 111 | 41 (36.94) | 0.97 (0.63-1.47) | 0.8686 | 111 | 43 (38.74) | 0.91 (0.61-1.35) | 0.6379 |
| **NLRP3** | C/C | 1114 | 700 | 1 |  | 335 | 168 (50.15) | 1 |  | 239 | 88 (36.82) | 1 |  | 239 | 99 (41.42) | 1 |  |
| **rs35829419** | A/C | 85 | 66 | 0.63 (0.41-0.97) | **0.037** | 29 | 9 (31.03) | 0.72 (0.37-1.42) | 0.3432 | 25 | 5 (20.00) | 0.57 (0.23-1.42) | 0.2276 | 25 | 6 (24.00) | 0.54 (0.23-1.22) | 0.1386 |
|  | A/A | 4 | 1 | 0.97 (0.09-10.11) | 0.9806 | - | - | - | - | - | - | - | - | - | - | - | - |
|  | C/A + A/A | 89 | 67 | 0.64 (0.42-0.98) | **0.04** | 29 | 9 (31.03) | 0.72 (0.37-1.42) | 0.3432 | 25 | 5 (20.00) | 0.57 (0.23-1.42) | 0.2276 | 25 | 6 (24.00) | 0.54 (0.23-1.22) | 0.1386 |
| **NLRP4** | G/G | 1109 | 703 | 1 |  | 324 | 159 (49.07) | 1 |  | 233 | 82 (35.19) | 1 |  | 233 | 94 (40.34) | 1 |  |
| **rs12462372** | A/G | 105 | 62 | 1.31 (0.87-1.96) | 0.1988 | 37 | 15 (40.54) | 0.81 (0.48-1.38) | 0.4419 | 29 | 8 (27.59) | 0.67 (0.32-1.40) | 0.2869 | 29 | 10 (34.48) | 0.79 (0.41-1.51) | 0.4687 |
|  | A/A | 2 | 5 | 0.34 (0.05-2.30) | 0.2678 | 1 | 0 (0.00) | 0.00 (0.00-.) | 0.9803 | - | - | - | - | - | - | - | - |
|  | A/G + A/A | 107 | 67 | 1.23 (0.83-1.82) | 0.3117 | 38 | 15 (39.47) | 0.79 (0.46-1.35) | 0.3881 | 29 | 8 (27.59) | 0.67 (0.32-1.40) | 0.2869 | 29 | 10 (34.48) | 0.79 (0.41-1.51) | 0.4687 |
| **NLRP4** | G/G | 1112 | 705 | 1 |  | 335 | 161 (48.06) | 1 |  | 246 | 86 (34.96) | 1 |  | 246 | 99 (40.24) | 1 |  |
| **rs17857373** | C/G | 93 | 56 | 1.01 (0.65-1.55) | 0.9771 | 30 | 16 (53.33) | 1.09 (0.65-1.84) | 0.7342 | 20 | 7 (35.00) | 0.72 (0.33-1.59) | 0.4231 | 20 | 8 (40.00) | 0.76 (0.37-1.58) | 0.465 |
|  | C/C | 4 | 3 | 0.40 (0.05-3.43) | 0.4053 | 2 | 1 (50.00) | 0.35 (0.05-2.58) | 0.3058 | 1 | 1 (100.00) | 86.87 (6.95-1085.85) | **0.0005** | 1 | 1 (100.00) | 12.47 (1.46-106.52) | **0.0211** |
|  | C/G + C/C | 97 | 59 | 0.97 (0.64-1.49) | 0.9065 | 32 | 17 (53.13) | 0.98 (0.59-1.62) | 0.9253 | 21 | 8 (38.10) | 0.82 (0.39-1.73) | 0.6045 | 21 | 9 (42.86) | 0.85 (0.43-1.70) | 0.6445 |
| **NLRP4** | A/A | 645 | 425 | 1 |  | 191 | 91 (47.64) | 1 |  | 135 | 45 (33.33) | 1 |  | 135 | 50 (37.04) | 1 |  |
| **rs302453** | A/T | 439 | 261 | 1.24 (0.97-1.59) | 0.0912 | 147 | 72 (48.98) | 1.21 (0.89-1.65) | 0.2324 | 112 | 41 (36.61) | 1.18 (0.77-1.82) | 0.4397 | 112 | 49 (43.75) | 1.25 (0.84-1.87) | 0.2737 |
|  | T/T | 82 | 58 | 0.77 (0.48-1.21) | 0.2558 | 17 | 10 (58.82) | 1.23 (0.63-2.38) | 0.5407 | 9 | 3 (33.33) | 1.12 (0.34-3.68) | 0.8552 | 9 | 3 (33.33) | 1.05 (0.32-3.40) | 0.9351 |
|  | A/A + T/T | 521 | 319 | 1.14 (0.90-1.45) | 0.2646 | 164 | 82 (50.00) | 1.21 (0.90-1.64) | 0.2116 | 121 | 44 (36.36) | 1.18 (0.77-1.80) | 0.4423 | 121 | 52 (42.98) | 1.24 (0.83-1.84) | 0.2913 |
| **NLRP4** | C/C | 489 | 319 | 1 |  | 142 | 71 (50.00) | 1 |  | 104 | 37 (35.58) | 1 |  | 104 | 41 (39.42) | 1 |  |
| **rs441827** | T/C | 533 | 316 | 1.20 (0.93-1.54) | 0.1587 | 170 | 77 (45.29) | 0.87 (0.63-1.20) | 0.3978 | 124 | 40 (32.26) | 1.14 (0.72-1.81) | 0.5812 | 124 | 47 (37.90) | 1.01 (0.65-1.55) | 0.9741 |
|  | T/T | 164 | 114 | 0.94 (0.66-1.34) | 0.733 | 44 | 23 (52.27) | 0.79 (0.49-1.27) | 0.3258 | 30 | 12 (40.00) | 1.49 (0.77-2.90) | 0.2407 | 30 | 15 (50.00) | 1.33 (0.73-2.42) | 0.3477 |
|  | C/T + T/T | 697 | 430 | 1.13 (0.89-1.43) | 0.3155 | 214 | 100 (46.73) | 0.85 (0.62-1.16) | 0.3008 | 154 | 52 (33.77) | 1.20 (0.78-1.87) | 0.4063 | 154 | 62 (40.26) | 1.07 (0.71-1.61) | 0.7331 |
| **NLRP5** | G/G | 684 | 457 | 1 |  | 197 | 88 (44.67) | 1 |  | 143 | 42 (29.37) | 1 |  | 143 | 48 (33.57) | 1 |  |
| **rs10409555** | A/G | 447 | 248 | 1.12 (0.87-1.43) | 0.3829 | 140 | 68 (48.57) | 1.20 (0.87-1.64) | 0.2719 | 104 | 39 (37.50) | 1.53 (0.98-2.38) | 0.0623 | 104 | 47 (45.19) | 1.56 (1.04-2.35) | **0.0317** |
|  | A/A | 73 | 50 | 1.30 (0.81-2.10) | 0.2769 | 25 | 18 (72.00) | 1.58 (0.93-2.69) | 0.0888 | 17 | 10 (58.82) | 3.04 (1.48-6.23) | **0.0024** | 17 | 10 (58.82) | 2.36 (1.17-4.78) | **0.017** |
|  | A/G + A/A | 520 | 298 | 1.14 (0.91-1.44) | 0.2598 | 165 | 86 (52.12) | 1.26 (0.93-1.70) | 0.1332 | 121 | 49 (40.50) | 1.69 (1.11-2.58) | **0.015** | 121 | 57 (47.11) | 1.66 (1.12-2.45) | **0.0116** |

| **NLRP5** | C/C | 920 | 591 | 1 |  | 268 | 122 (45.52) | 1 |  | 195 | 61 (31.28) | 1 |  | 195 | 69 (35.38) | 1 |  |
| --- | --- | --- | --- | --- | --- | --- | --- | --- | --- | --- | --- | --- | --- | --- | --- | --- | --- |
| **rs12462795** | C/G | 261 | 171 | 0.96 (0.73-1.27) | 0.7876 | 90 | 47 (52.22) | 1.28 (0.91-1.81) | 0.1528 | 68 | 28 (41.18) | 1.71 (1.07-2.74) | **0.0253** | 68 | 34 (50.00) | 1.80 (1.17-2.76) | **0.0071** |
|  | G/G | 20 | 10 | 1.79 (0.69-4.64) | 0.228 | 6 | 5 (83.33) | 2.79 (1.13-6.90) | **0.0261** | 3 | 2 (66.67) | 3.05 (0.73-12.74) | 0.1254 | 3 | 2 (66.67) | 2.72 (0.66-11.29) | 0.1671 |
|  | C/G + G/G | 281 | 181 | 1.79 (0.69-4.64) | 0.9819 | 96 | 52 (54.17) | 1.36 (0.97-1.89) | 0.0714 | 71 | 30 (42.25) | 1.77 (1.12-2.81) | **0.0145** | 71 | 36 (50.70) | 1.84 (1.21-2.79) | **0.0045** |
| **NLRP5** | T/T | 852 | 543 | 1 |  | 253 | 118 (46.64) | 1 |  | 184 | 57 (30.98) | 1 |  | 184 | 64 (34.78) | 1 |  |
| **rs16986899** | C/T | 303 | 201 | 0.93 (0.72-1.21) | 0.5942 | 99 | 54 (54.55) | 1.21 (0.87-1.67) | 0.2591 | 73 | 33 (45.21) | 1.70 (1.10-2.64) | **0.0172** | 73 | 39 (53.42) | 1.74 (1.17-2.61) | **0.0068** |
|  | C/C | 40 | 22 | 1.36 (0.69-2.66) | 0.3703 | 8 | 2 (25.00) | 0.27 (0.07-1.13) | 0.0733 | 4 | 0 (0.00) | 0.00 (0.00-.) | 0.9796 | 4 | 0 (0.00) | 0.00 (0.00-.) | 0.978 |
|  | C/T + C/C | 343 | 223 | 0.97 (0.75-1.25) | 0.8114 | 107 | 56 (52.34) | 1.08 (0.78-1.49) | 0.6443 | 77 | 33 (42.86) | 1.58 (1.02-2.44) | **0.0401** | 77 | 39 (50.65) | 1.61 (1.08-2.40) | **0.0202** |
| **NLRP5** | G/G | 983 | 626 | 1 |  | 301 | 146 (48.50) | 1 |  | 222 | 81 (36.49) | 1 |  | 222 | 89 (40.09) | 1 |  |
| **rs471979** | C/G | 224 | 127 | 1.27 (0.94-1.72) | 0.1235 | 67 | 32 (47.76) | 0.96 (0.65-1.41) | 0.8397 | 48 | 14 (29.17) | 0.78 (0.44-1.39) | 0.4029 | 48 | 20 (41.67) | 1.05 (0.64-1.70) | 0.8594 |
|  | C/C | 10 | 13 | 0.73 (0.28-1.91) | 0.5267 | 2 | 1 (50.00) | 1.17 (0.16-8.54) | 0.8762 | 1 | 0 (0.00) | 0.00 (0.00-.) | 0.9825 | 1 | 0 (0.00) | 0.00 (0.00-.) | 0.9819 |
|  | C/G + C/C | 234 | 140 | 1.22 (0.91-1.63) | 0.1918 | 69 | 33 (47.83) | 0.97 (0.66-1.42) | 0.861 | 49 | 14 (28.57) | 0.76 (0.43-1.35) | 0.3474 | 49 | 20 (40.82) | 1.02 (0.62-1.66) | 0.9501 |
| **NLRP6** | T/T | 927 | 630 | 1 |  | 272 | 135 (49.63) | 1 |  | 200 | 72 (36.00) | 1 |  | 200 | 82 (41.00) | 1 |  |
| **rs56159585** | T/A | 221 | 118 | 1.30 (0.95-1.77) | 0.1047 | 75 | 32 (42.67) | 1.00 (0.67-1.47) | 0.9851 | 57 | 17 (29.82) | 0.91 (0.53-1.54) | 0.7168 | 57 | 20 (35.09) | 0.85 (0.52-1.38) | 0.5084 |
|  | A/A | 19 | 7 | 0.98 (0.37-2.56) | 0.9629 | 7 | 3 (42.86) | 1.08 (0.34-3.46) | 0.9014 | 4 | 1 (25.00) | 4.03 (0.51-31.68) | 0.1848 | 4 | 1 (25.00) | 1.62 (0.21-12.48) | 0.6416 |
|  | T/A + A/A | 240 | 125 | 1.27 (0.94-1.71) | 0.1249 | 82 | 35 (42.68) | 1.00 (0.69-1.46) | 0.988 | 61 | 18 (29.51) | 0.95 (0.56-1.60) | 0.8457 | 61 | 21 (34.43) | 0.87 (0.54-1.40) | 0.5644 |
| **NLRP6** | G/G | 924 | 629 | 1 |  | 274 | 134 (48.91) | 1 |  | 204 | 72 (35.29) | 1 |  | 204 | 82 (40.20) | 1 |  |
| **rs6421985** | T/G | 252 | 128 | 1.36 (1.01-1.83) | **0.0421** | 82 | 37 (45.12) | 0.99 (0.69-1.44) | 0.9743 | 56 | 17 (30.36) | 1.04 (0.61-1.78) | 0.8867 | 56 | 21 (37.50) | 1.05 (0.65-1.70) | 0.8538 |
|  | T/T | - | - | - | - | - | - | - | - | - | - | - | - | - | - | - | - |
|  | T/G + T/T | 252 | 128 | 1.36 (1.01-1.83) | **0.0421** | 82 | 37 (45.12) | 0.99 (0.69-1.44) | 0.9743 | 56 | 17 (30.36) | 1.04 (0.61-1.78) | 0.8867 | 56 | 21 (37.50) | 1.05 (0.65-1.70) | 0.8538 |
| **NLRP6** | C/C | 776 | 488 | 1 |  | 226 | 104 (46.02) | 1 |  | 163 | 51 (31.29) | 1 |  | 163 | 58 (35.58) | 1 |  |
| **rs77447196** | G/C | 342 | 231 | 0.88 (0.68-1.13) | 0.3142 | 107 | 55 (51.40) | 1.05 (0.75-1.47) | 0.7798 | 82 | 34 (41.46) | 1.31 (0.84-2.04) | 0.234 | 82 | 40 (48.78) | 1.43 (0.94-2.16) | 0.0945 |
|  | G/G | 51 | 35 | 0.96 (0.56-1.66) | 0.89 | 20 | 9 (45.00) | 1.37 (0.69-2.72) | 0.3731 | 15 | 6 (40.00) | 1.39 (0.59-3.26) | 0.4549 | 15 | 6 (40.00) | 1.26 (0.54-2.93) | 0.5996 |
|  | G/C + G/G | 393 | 266 | 0.89 (0.70-1.13) | 0.3404 | 127 | 64 (50.39) | 1.09 (0.79-1.50) | 0.6055 | 97 | 40 (41.24) | 1.32 (0.87-2.02) | 0.1969 | 97 | 46 (47.42) | 1.40 (0.94-2.08) | 0.0976 |
| **NLRP7** | C/C | 952 | 624 | 1 |  | 287 | 136 (47.39) | 1 |  | 210 | 72 (34.29) | 1 |  | 210 | 85 (40.48) | 1 |  |
| **rs61747414** | C/T | 226 | 131 | 1.02 (0.76-1.38) | 0.8969 | 71 | 36 (50.70) | 0.94 (0.65-1.36) | 0.7485 | 52 | 18 (34.62) | 0.86 (0.51-1.44) | 0.5609 | 52 | 18 (34.62) | 0.77 (0.46-1.29) | 0.3182 |
|  | T/T | 15 | 13 | 0.98 (0.37-2.61) | 0.966 | 2 | 2 (100.00) | 3.29 (0.80-13.59) | 0.1001 | 2 | 2 (100.00) | 3.35 (0.80-14.02) | 0.098 | 2 | 2 (100.00) | 3.11 (0.74-13.13) | 0.1223 |
|  | C/T + T/T | 241 | 144 | 1.02 (0.76-1.36) | 0.9096 | 73 | 38 (52.05) | 0.98 (0.68-1.40) | 0.9018 | 54 | 20 (37.04) | 0.93 (0.56-1.53) | 0.7644 | 54 | 20 (37.04) | 0.83 (0.51-1.36) | 0.4683 |
| **NLRP7** | G/G | 813 | 519 | 1 |  | 237 | 119 (50.21) | 1 |  | 179 | 69 (38.55) | 1 |  | 179 | 76 (42.46) | 1 |  |
| **rs7359934** | A/G | 353 | 189 | 1.20 (0.92-1.56) | 0.1765 | 105 | 49 (46.67) | 0.86 (0.61-1.20) | 0.3717 | 72 | 21 (29.17) | 0.83 (0.50-1.35) | 0.4445 | 72 | 26 (36.11) | 0.82 (0.52-1.29) | 0.3928 |
|  | A/A | 38 | 37 | 0.59 (0.33-1.04) | 0.0667 | 12 | 5 (41.67) | 1.25 (0.50-3.09) | 0.6338 | 8 | 1 (12.50) | 0.28 (0.04-2.01) | 0.2036 | 8 | 2 (25.00) | 0.50 (0.12-2.07) | 0.3407 |
|  | A/G + A/A | 391 | 226 | 1.09 (0.85-1.39) | 0.5134 | 117 | 54 (46.15) | 0.88 (0.64-1.22) | 0.4583 | 80 | 22 (27.50) | 0.76 (0.47-1.23) | 0.2668 | 80 | 28 (35.00) | 0.79 (0.51-1.22) | 0.2837 |
| **NLRP8** | G/G | 732 | 493 | 1 |  | 230 | 106 (46.09) | 1 |  | 170 | 58 (34.12) | 1 |  | 170 | 68 (40.00) | 1 |  |
| **rs306457** | C/G | 415 | 235 | 1.13 (0.88-1.45) | 0.3241 | 115 | 61 (53.04) | 1.25 (0.91-1.72) | 0.169 | 82 | 30 (36.59) | 1.08 (0.69-1.68) | 0.7456 | 82 | 33 (40.24) | 1.02 (0.67-1.54) | 0.9392 |
|  | C/C | 63 | 26 | 2.01 (1.09-3.72) | **0.0257** | 22 | 11 (50.00) | 1.01 (0.54-1.89) | 0.9724 | 15 | 6 (40.00) | 1.00 (0.41-2.40) | 0.997 | 15 | 7 (46.67) | 1.23 (0.55-2.74) | 0.6142 |
|  | C/G + C/C | 478 | 261 | 1.20 (0.95-1.53) | 0.1267 | 137 | 72 (52.55) | 1.21 (0.89-1.63) | 0.2231 | 97 | 36 (37.11) | 1.06 (0.70-1.63) | 0.7741 | 97 | 40 (41.24) | 1.05 (0.70-1.55) | 0.8233 |
| **NLRP8** | G/G | 421 | 277 | 1 |  | 124 | 58 (46.77) | 1 |  | 92 | 30 (32.61) | 1 |  | 92 | 35 (38.04) | 1 |  |
| **rs306481** | A/G | 545 | 320 | 1.28 (0.99-1.67) | 0.0599 | 167 | 79 (47.31) | 0.90 (0.64-1.27) | 0.5461 | 119 | 40 (33.61) | 1.02 (0.63-1.63) | 0.9499 | 119 | 47 (39.50) | 1.03 (0.66-1.60) | 0.9011 |
|  | A/A | 202 | 141 | 0.87 (0.63-1.22) | 0.4298 | 59 | 29 (49.15) | 1.06 (0.67-1.67) | 0.814 | 46 | 18 (39.13) | 1.12 (0.62-2.02) | 0.712 | 46 | 20 (43.48) | 1.14 (0.66-1.98) | 0.6434 |
|  | A/G + A/A | 747 | 461 | 1.15 (0.90-1.46) | 0.2594 | 226 | 108 (47.79) | 0.94 (0.68-1.29) | 0.6855 | 165 | 58 (35.15) | 1.04 (0.67-1.63) | 0.8458 | 165 | 67 (40.61) | 1.06 (0.70-1.60) | 0.7831 |
| **NLRP8** | G/G | 334 | 202 | 1 |  | 93 | 44 (47.31) | 1 |  | 69 | 24 (34.78) | 1 |  | 69 | 26 (37.68) | 1 |  |
| **rs306496** | G/A | 601 | 341 | 1.24 (0.94-1.63) | 0.1282 | 180 | 92 (51.11) | 1.11 (0.77-1.59) | 0.5724 | 129 | 50 (38.76) | 1.23 (0.75-2.01) | 0.4119 | 129 | 60 (46.51) | 1.45 (0.91-2.31) | 0.1212 |
|  | A/A | 265 | 180 | 0.88 (0.63-1.21) | 0.4296 | 86 | 40 (46.51) | 0.91 (0.59-1.40) | 0.6768 | 64 | 20 (31.25) | 0.69 (0.38-1.25) | 0.2172 | 64 | 21 (32.81) | 0.77 (0.43-1.37) | 0.3698 |
|  | A/G + A/A | 866 | 521 | 1.11 (0.86-1.43) | 0.4326 | 266 | 132 (49.62) | 1.04 (0.74-1.46) | 0.8191 | 193 | 70 (36.27) | 1.00 (0.63-1.59) | 0.9953 | 193 | 81 (41.97) | 1.17 (0.75-1.82) | 0.4941 |
| **NLRP8** | T/T | 429 | 289 | 1 |  | 123 | 57 (46.34) | 1 |  | 92 | 30 (32.61) | 1 |  | 92 | 39 (42.39) | 1 |  |
| **rs306507** | C/T | 575 | 353 | 1.16 (0.90-1.50) | 0.2406 | 172 | 86 (50.00) | 1.06 (0.76-1.49) | 0.7196 | 124 | 46 (37.10) | 1.08 (0.68-1.72) | 0.7466 | 124 | 49 (39.52) | 0.87 (0.57-1.34) | 0.535 |
|  | C/C | 193 | 114 | 1.15 (0.80-1.63) | 0.4525 | 65 | 30 (46.15) | 0.93 (0.59-1.45) | 0.7367 | 46 | 14 (30.43) | 0.79 (0.41-1.50) | 0.4695 | 46 | 16 (34.78) | 0.74 (0.41-1.34) | 0.328 |
|  | C/T + C/C | 768 | 467 | 1.16 (0.91-1.47) | 0.2258 | 237 | 116 (48.95) | 1.02 (0.74-1.41) | 0.883 | 170 | 60 (35.29) | 1.00 (0.64-1.56) | 0.9913 | 170 | 65 (38.24) | 0.84 (0.56-1.25) | 0.3915 |

| **NLRP8** | G/G | 1032 | 675 | 1 |  | 312 | 148 (47.44) | 1 |  | 232 | 79 (34.05) | 1 |  | 232 | 91 (39.22) | 1 |  |
| --- | --- | --- | --- | --- | --- | --- | --- | --- | --- | --- | --- | --- | --- | --- | --- | --- | --- |
| **rs41481648** | G/A | 179 | 98 | 1.14 (0.82-1.59) | 0.4333 | 53 | 30 (56.60) | 1.25 (0.84-1.86) | 0.2738 | 35 | 16 (45.71) | 1.76 (1.02-3.04) | **0.0425** | 35 | 18 (51.43) | 1.54 (0.92-2.57) | 0.1015 |
|  | A/A | 5 | 5 | 1.06 (0.22-5.12) | 0.9465 | 2 | 0 (0.00) | 0.00 (0.00-.) | 0.9801 | 2 | 0 (0.00) | 0.00 (0.00-.) | 0.9819 | 2 | 0 (0.00) | 0.00 (0.00-.) | 0.978 |
|  | G/A + G/G | 184 | 103 | 1.14 (0.82-1.58) | 0.436 | 55 | 30 (54.55) | 1.20 (0.81-1.79) | 0.3642 | 37 | 16 (43.24) | 1.63 (0.95-2.82) | 0.0778 | 37 | 18 (48.65) | 1.44 (0.86-2.40) | 0.1672 |
| **NLRP8** | A/A | 994 | 614 | 1 |  | 296 | 146 (49.32) | 1 |  | 214 | 77 (35.98) | 1 |  | 214 | 89 (41.59) | 1 |  |
| **rs61740015** | A/G | 218 | 141 | 0.90 (0.67-1.21) | 0.4864 | 68 | 31 (45.59) | 1.01 (0.69-1.50) | 0.9461 | 50 | 16 (32.00) | 1.00 (0.58-1.73) | 0.9918 | 50 | 18 (36.00) | 0.89 (0.53-1.48) | 0.6415 |
|  | G/G | 14 | 11 | 0.54 (0.18-1.59) | 0.2627 | 7 | 3 (42.86) | 0.76 (0.24-2.41) | 0.6421 | 6 | 2 (33.33) | 0.53 (0.13-2.19) | 0.3796 | 6 | 2 (33.33) | 0.53 (0.13-2.16) | 0.3748 |
|  | G/A + G/G | 232 | 152 | 0.87 (0.65-1.17) | 0.361 | 75 | 34 (45.33) | 0.98 (0.68-1.43) | 0.9342 | 56 | 18 (32.14) | 0.90 (0.54-1.52) | 0.7056 | 56 | 20 (35.71) | 0.83 (0.51-1.35) | 0.4479 |
| **NLRP8** | A/A | 1092 | 689 | 1 |  | 325 | 159 (48.92) | 1 |  | 238 | 87 (36.55) | 1 |  | 238 | 99 (41.60) | 1 |  |
| **rs7259764** | A/G | 123 | 73 | 1.15 (0.78-1.71) | 0.4735 | 41 | 19 (46.34) | 0.98 (0.61-1.58) | 0.9229 | 30 | 8 (26.67) | 0.69 (0.33-1.44) | 0.3239 | 30 | 10 (33.33) | 0.77 (0.40-1.48) | 0.4355 |
|  | G/G | 2 | 4 | 0.25 (0.03-2.05) | 0.195 | - | - | - | - | - | - | - | - | - | - | - | - |
|  | A/G + A/A | 125 | 77 | 1.10 (0.75-1.61) | 0.6398 | 41 | 19 (46.34) | 0.98 (0.61-1.58) | 0.9229 | 30 | 8 (26.67) | 0.69 (0.33-1.44) | 0.3239 | 30 | 10 (33.33) | 0.77 (0.40-1.48) | 0.4355 |
| **NLRP11** | G/G | 522 | 312 | 1 |  | 163 | 74 (45.40) | 1 |  | 124 | 43 (34.68) | 1 |  | 124 | 50 (40.32) | 1 |  |
| **rs12461110** | A/G | 484 | 326 | 0.75 (0.58-0.96) | **0.0227** | 133 | 68 (51.13) | 1.10 (0.79-1.53) | 0.5786 | 89 | 28 (31.46) | 0.97 (0.60-1.56) | 0.8846 | 89 | 31 (34.83) | 0.81 (0.51-1.27) | 0.3503 |
|  | A/A | 181 | 126 | 0.76 (0.54-1.07) | 0.1152 | 61 | 29 (47.54) | 1.09 (0.71-1.69) | 0.6917 | 49 | 19 (38.78) | 0.91 (0.52-1.59) | 0.7444 | 49 | 22 (44.90) | 0.94 (0.56-1.57) | 0.7987 |
|  | A/G + A/A | 665 | 452 | 0.75 (0.59-0.95) | **0.0159** | 194 | 97 (50.00) | 1.10 (0.81-1.49) | 0.5525 | 138 | 47 (34.06) | 0.94 (0.62-1.44) | 0.7869 | 138 | 53 (38.41) | 0.85 (0.57-1.27) | 0.4315 |
| **NLRP11** | A/A | 1070 | 662 | 1 |  | 325 | 159 (48.92) | 1 |  | 238 | 84 (35.29) | 1 |  | 238 | 97 (40.76) | 1 |  |
| **rs299163** | A/C | 134 | 91 | 0.94 (0.66-1.34) | 0.7422 | 34 | 16 (47.06) | 1.18 (0.70-1.99) | 0.5285 | 26 | 10 (38.46) | 1.19 (0.61-2.31) | 0.6057 | 26 | 10 (38.46) | 0.84 (0.44-1.63) | 0.6148 |
|  | C/C | 7 | 12 | 0.21 (0.06-0.68) | **0.0097** | 2 | 0 (0.00) | 0.00 (0.00-.) | 0.9776 | 2 | 0 (0.00) | 0.00 (0.00-.) | 0.9858 | 2 | 0 (0.00) | 0.00 (0.00-.) | 0.9843 |
|  | A/C + C/C | 141 | 103 | 0.83 (0.59-1.17) | 0.2954 | 36 | 16 (44.44) | 1.12 (0.67-1.89) | 0.6604 | 28 | 10 (35.71) | 1.10 (0.57-2.14) | 0.7803 | 28 | 10 (35.71) | 0.79 (0.41-1.52) | 0.4729 |
| **NLRP12** | C/C | 729 | 470 | 1 |  | 225 | 112 (49.78) | 1 |  | 161 | 60 (37.27) | 1 |  | 161 | 71 (44.10) | 1 |  |
| **rs34436714** | A/C | 388 | 244 | 1.05 (0.81-1.35) | 0.7229 | 113 | 50 (44.25) | 0.93 (0.66-1.30) | 0.6658 | 83 | 23 (27.71) | 0.72 (0.45-1.17) | 0.1898 | 83 | 26 (31.33) | 0.67 (0.43-1.06) | 0.0855 |
|  | A/A | 65 | 30 | 1.48 (0.85-2.59) | 0.1675 | 16 | 6 (37.50) | 0.76 (0.33-1.73) | 0.511 | 13 | 3 (23.08) | 0.51 (0.16-1.65) | 0.2627 | 13 | 3 (23.08) | 0.47 (0.15-1.49) | 0.1985 |
|  | A/C + A/A | 453 | 274 | 1.10 (0.86-1.39) | 0.4591 | 129 | 56 (43.41) | 0.91 (0.65-1.26) | 0.5542 | 96 | 26 (27.08) | 0.69 (0.43-1.10) | 0.1175 | 96 | 29 (30.21) | 0.64 (0.42-0.99) | **0.0464** |
| **NLRP12** | G/G | 1047 | 655 | 1 |  | 318 | 154 (48.43) | 1 |  | 235 | 83 (35.32) | 1 |  | 235 | 95 (40.43) | 1 |  |
| **rs34971363** | C/G | 150 | 102 | 0.97 (0.69-1.36) | 0.8439 | 40 | 18 (45.00) | 0.81 (0.50-1.32) | 0.4014 | 28 | 9 (32.14) | 0.79 (0.40-1.58) | 0.5122 | 28 | 11 (39.29) | 0.85 (0.46-1.60) | 0.6189 |
|  | C/C | 13 | 4 | 1.40 (0.38-5.21) | 0.6162 | 3 | 2 (66.67) | 1.84 (0.44-7.64) | 0.4024 | 1 | 0 (0.00) | 0.00 (0.00-.) | 0.9833 | 1 | 0 (0.00) | 0.00 (0.00-.) | 0.9821 |
|  | C/G + C/C | 163 | 106 | 0.99 (0.71-1.38) | 0.9407 | 43 | 20 (46.51) | 0.86 (0.54-1.37) | 0.5208 | 29 | 9 (31.03) | 0.71 (0.36-1.43) | 0.339 | 29 | 11 (37.93) | 0.79 (0.42-1.48) | 0.4561 |
| **NLRP13** | T/T | 1008 | 656 | 1 |  | 295 | 136 (46.10) | 1 |  | 220 | 72 (32.73) | 1 |  | 220 | 84 (38.18) | 1 |  |
| **rs17711239** | C/T | 205 | 92 | 1.33 (0.95-1.86) | 0.0943 | 65 | 38 (58.46) | 1.12 (0.78-1.62) | 0.5277 | 45 | 21 (46.67) | 1.55 (0.95-2.55) | 0.0819 | 45 | 23 (51.11) | 1.53 (0.96-2.43) | 0.0761 |
|  | C/C | 6 | 14 | 0.34 (0.11-0.99) | **0.0487** | 4 | 0 (0.00) | 0.00 (0.00-.) | 0.9774 | 3 | 0 (0.00) | 0.00 (0.00-.) | 0.982 | 3 | 0 (0.00) | 0.00 (0.00-.) | 0.9804 |
|  | C/T + C/C | 211 | 106 | 1.18 (0.86-1.62) | 0.3056 | 69 | 38 (55.07) | 1.05 (0.73-1.51) | 0.8039 | 48 | 21 (43.75) | 1.40 (0.86-2.30) | 0.1794 | 48 | 23 (47.92) | 1.39 (0.87-2.21) | 0.1713 |
| **NLRP13** | C/C | 419 | 297 | 1 |  | 133 | 60 (45.11) | 1 |  | 102 | 33 (32.35) | 1 |  | 102 | 36 (35.29) | 1 |  |
| **rs303997** | C/T | 564 | 334 | 1.37 (1.06-1.77) | **0.0152** | 163 | 86 (52.76) | 1.15 (0.82-1.61) | 0.4073 | 116 | 46 (39.66) | 1.43 (0.91-2.25) | 0.1181 | 116 | 55 (47.41) | 1.46 (0.96-2.23) | 0.0799 |
|  | T/T | 212 | 125 | 1.54 (1.10-2.16) | **0.0128** | 63 | 26 (41.27) | 0.77 (0.48-1.22) | 0.2652 | 47 | 13 (27.66) | 0.96 (0.50-1.84) | 0.8934 | 47 | 15 (31.91) | 0.97 (0.53-1.78) | 0.918 |
|  | C/T + T/T | 776 | 459 | 1.42 (1.11-1.80) | **0.0045** | 226 | 112 (49.56) | 1.03 (0.75-1.42) | 0.8477 | 163 | 59 (36.20) | 1.30 (0.84-2.00) | 0.2382 | 163 | 70 (42.94) | 1.32 (0.88-1.98) | 0.1797 |
| **NLRP14** | A/A | 455 | 272 | 1 |  | 135 | 64 (47.41) | 1 |  | 99 | 32 (32.32) | 1 |  | 99 | 40 (40.40) | 1 |  |
| **rs10839708** | A/G | 561 | 368 | 0.90 (0.70-1.16) | 0.4334 | 163 | 74 (45.40) | 0.89 (0.64-1.25) | 0.5147 | 124 | 44 (35.48) | 1.12 (0.71-1.77) | 0.6304 | 124 | 49 (39.52) | 0.95 (0.63-1.45) | 0.8297 |
|  | G/G | 187 | 116 | 1.08 (0.76-1.53) | 0.6717 | 61 | 36 (59.02) | 1.24 (0.82-1.88) | 0.301 | 40 | 17 (42.50) | 1.39 (0.77-2.52) | 0.2744 | 40 | 18 (45.00) | 1.18 (0.67-2.07) | 0.5614 |
|  | A/G + A/A | 748 | 484 | 0.94 (0.74-1.20) | 0.6353 | 224 | 110 (49.11) | 0.98 (0.72-1.34) | 0.9213 | 164 | 61 (37.20) | 1.18 (0.77-1.82) | 0.4447 | 164 | 67 (40.85) | 1.01 (0.68-1.49) | 0.9764 |
| **NLRP14** | C/C | 748 | 488 | 1 |  | 220 | 106 (48.18) | 1 |  | 161 | 56 (34.78) | 1 |  | 161 | 66 (40.99) | 1 |  |
| **rs17280682** | C/T | 413 | 238 | 0.98 (0.76-1.25) | 0.8656 | 128 | 61 (47.66) | 0.82 (0.59-1.13) | 0.2213 | 92 | 32 (34.78) | 1.02 (0.65-1.59) | 0.9335 | 92 | 36 (39.13) | 0.94 (0.62-1.42) | 0.7631 |
|  | T/T | 54 | 38 | 0.99 (0.56-1.72) | 0.959 | 20 | 11 (55.00) | 1.36 (0.73-2.56) | 0.334 | 15 | 6 (40.00) | 1.18 (0.51-2.76) | 0.7006 | 15 | 6 (40.00) | 0.85 (0.37-1.97) | 0.7044 |
|  | C/T + T/T | 467 | 276 | 0.98 (0.77-1.24) | 0.8652 | 148 | 72 (48.65) | 0.87 (0.64-1.18) | 0.3795 | 107 | 38 (35.51) | 1.04 (0.68-1.59) | 0.847 | 107 | 42 (39.25) | 0.92 (0.62-1.37) | 0.6955 |
| **NLRC5** | C/C | 645 | 399 | 1 |  | 192 | 101 (52.60) | 1 |  | 133 | 53 (39.85) | 1 |  | 133 | 61 (45.86) | 1 |  |
| **rs289723** | A/C | 476 | 284 | 1.07 (0.84-1.37) | 0.5894 | 149 | 65 (43.62) | 0.87 (0.64-1.20) | 0.3999 | 113 | 33 (29.20) | 0.72 (0.46-1.11) | 0.1386 | 113 | 37 (32.74) | 0.61 (0.41-0.93) | **0.0208** |
|  | A/A | 92 | 67 | 1.01 (0.66-1.54) | 0.9764 | 27 | 11 (40.74) | 0.99 (0.53-1.86) | 0.9777 | 23 | 8 (34.78) | 1.13 (0.53-2.41) | 0.7449 | 23 | 10 (43.48) | 1.04 (0.53-2.04) | 0.9179 |
|  | A/C + A/A | 568 | 351 | 1.06 (0.84-1.34) | 0.6335 | 176 | 76 (43.18) | 0.89 (0.66-1.20) | 0.4417 | 136 | 41 (30.15) | 0.77 (0.51-1.17) | 0.2194 | 136 | 47 (34.56) | 0.67 (0.46-0.99) | **0.044** |
| **NLRC5** | G/G | 858 | 537 | 1 |  | 258 | 132 (51.16) | 1 |  | 187 | 72 (38.50) | 1 |  | 187 | 82 (43.85) | 1 |  |
| **rs3751710** | G/A | 313 | 197 | 1.04 (0.80-1.35) | 0.782 | 91 | 40 (43.96) | 0.92 (0.64-1.32) | 0.6504 | 68 | 21 (30.88) | 0.81 (0.50-1.33) | 0.4075 | 68 | 24 (35.29) | 0.76 (0.48-1.21) | 0.2469 |
|  | A/A | 45 | 25 | 1.20 (0.65-2.20) | 0.5623 | 16 | 4 (25.00) | 0.81 (0.29-2.21) | 0.6747 | 13 | 1 (7.69) | 0.21 (0.03-1.50) | 0.1197 | 13 | 2 (15.38) | 0.37 (0.09-1.49) | 0.1605 |
|  | G/A + A/A | 358 | 222 | 1.06 (0.82-1.36) | 0.6663 | 107 | 44 (41.12) | 0.91 (0.64-1.28) | 0.5892 | 81 | 22 (27.16) | 0.72 (0.44-1.17) | 0.1797 | 81 | 26 (32.10) | 0.70 (0.45-1.10) | 0.1204 |
| **NLRC5** | A/A | 1039 | 667 | 1 |  | 312 | 153 (49.04) | 1 |  | 228 | 82 (35.96) | 1 |  | 228 | 93 (40.79) | 1 |  |
| **rs7185320** | A/G | 152 | 101 | 0.87 (0.62-1.23) | 0.4285 | 48 | 24 (50.00) | 0.98 (0.64-1.52) | 0.9361 | 34 | 12 (35.29) | 0.90 (0.48-1.67) | 0.7337 | 34 | 15 (44.12) | 1.11 (0.64-1.95) | 0.7067 |
|  | G/G | 13 | 2 | 3.28 (0.63-17.06) | 0.1581 | 3 | 1 (33.33) | 0.91 (0.12-6.66) | 0.9254 | 2 | 0 (0.00) | 0.00 (0.00-.) | 0.9841 | 2 | 0 (0.00) | 0.00 (0.00-.) | 0.9831 |
|  | GT+GG | 165 | 103 | 0.93 (0.66-1.30) | 0.6512 | 51 | 25 (49.02) | 0.98 (0.64-1.50) | 0.9231 | 36 | 12 (33.33) | 0.82 (0.45-1.52) | 0.5379 | 36 | 15 (41.67) | 1.01 (0.58-1.76) | 0.9701 |
| **NLRC5** | C/C | 902 | 542 | 1 |  | 282 | 128 (45.39) | 1 |  | 204 | 64 (31.37) | 1 |  | 204 | 73 (35.78) | 1 |  |
| **rs74439742** | C/T | 272 | 206 | 0.79 (0.61-1.03) | 0.0853 | 75 | 43 (57.33) | 1.42 (1.00-2.00) | **0.0501** | 56 | 26 (46.43) | 1.67 (1.05-2.65) | **0.0304** | 56 | 31 (55.36) | 1.63 (1.06-2.49) | **0.0262** |
|  | T/T | 30 | 20 | 0.88 (0.39-1.96) | 0.7466 | 10 | 7 (70.00) | 2.39 (1.10-5.19) | **0.0272** | 7 | 4 (57.14) | 3.07 (1.07-8.80) | **0.0368** | 7 | 4 (57.14) | 2.71 (0.96-7.67) | 0.061 |
|  | C/T + T/T | 302 | 226 | 0.80 (0.62-1.03) | 0.0867 | 85 | 50 (58.82) | 1.50 (1.08-2.09) | **0.0157** | 63 | 30 (47.62) | 1.77 (1.13-2.75) | **0.0121** | 63 | 35 (55.56) | 1.70 (1.13-2.57) | **0.0116** |
| **NLRC5** | T/T | 967 | 634 | 1 |  | 290 | 142 (48.97) | 1 |  | 211 | 74 (35.07) | 1 |  | 211 | 86 (40.76) | 1 |  |
| **rs28438857** | C/T | 213 | 125 | 1.10 (0.81-1.50) | 0.5307 | 69 | 31 (44.93) | 0.84 (0.57-1.24) | 0.378 | 52 | 18 (34.62) | 0.91 (0.54-1.52) | 0.7073 | 52 | 20 (38.46) | 0.92 (0.56-1.51) | 0.7418 |
|  | C/C | 15 | 11 | 1.28 (0.43-3.77) | 0.6558 | 7 | 4 (57.14) | 1.76 (0.65-4.81) | 0.269 | 4 | 1 (25.00) | 1.07 (0.15-7.85) | 0.9439 | 4 | 1 (25.00) | 0.76 (0.10-5.53) | 0.7851 |
|  | C/T + C/C | 228 | 136 | 1.11 (0.82-1.51) | 0.4804 | 76 | 35 (46.05) | 0.89 (0.61-1.29) | 0.5483 | 56 | 19 (33.93) | 0.91 (0.55-1.52) | 0.7256 | 56 | 21 (37.50) | 0.91 (0.56-1.47) | 0.7036 |
| **NLRC5** | T/T | 694 | 417 | 1 |  | 209 | 96 (45.93) | 1 |  | 152 | 49 (32.24) | 1 |  | 152 | 57 (37.50) | 1 |  |
| **rs78176773** | C/T | 421 | 266 | 0.93 (0.73-1.19) | 0.5521 | 128 | 66 (51.56) | 1.11 (0.81-1.52) | 0.5161 | 91 | 34 (37.36) | 1.14 (0.73-1.76) | 0.5685 | 91 | 39 (42.86) | 1.10 (0.73-1.66) | 0.6466 |
|  | C/C | 85 | 60 | 0.82 (0.53-1.27) | 0.3715 | 30 | 15 (50.00) | 1.16 (0.67-2.02) | 0.5897 | 25 | 10 (40.00) | 1.09 (0.55-2.15) | 0.8138 | 25 | 11 (44.00) | 1.09 (0.57-2.08) | 0.8012 |
|  | C/T + C/C | 506 | 326 | 0.91 (0.72-1.14) | 0.4103 | 158 | 81 (51.27) | 1.12 (0.83-1.51) | 0.4575 | 116 | 44 (37.93) | 1.12 (0.75-1.69) | 0.5739 | 116 | 50 (43.10) | 1.10 (0.75-1.61) | 0.6324 |

**Table D: mRNA Expression for the most promising candidate genes:** Study data and reported somatic mutations for CRC-associated NLRs.

| Gene |  | Gut-related tissues | | | | Immune cells | | | Other | Somatic Mutations in Colon Adenocarcinoma | |
| --- | --- | --- | --- | --- | --- | --- | --- | --- | --- | --- | --- |
|  |  | Duodenum | Ileum | Colon | Rectum | Mono | MoMac | PMN |  | COSMIC Somatic mutations^a^ | IntOGen Driver mutations^b^ |
| NLRP2 | This study | - | + | + | - | - | + | - | N/A | + sML (4.56%) | - |
| NLRP3 | This study | + | + | + | + | +++ | ++ | +++ | N/A | + sML (5.93%), sMS (2.5%) | - |
| NLRP5 | This study | - | - | - | - | - | - | - | +++ (Ovary) | + sML (6.18%) | - |
| NLRP13 | This study | - | - | - | - | - | - | - | +++ (Ovary) | - sML (5.51%) | - |
| NLRC5 | This study | + | + | + | ++ | N/A | N/A | N/A | N/A | +++ sML (6.7%) | - |

a somatic mutations reported in COSMIC database for: sML intestine (~1000 samples recorded and analysed); sMS small intestine (40 samples). Percentage of mutated cases out of the total of samples in COSMIC is given in brackets. <http://cancer.sanger.ac.uk/cosmic>. b Driver mutations reported by IntOGen <https://www.intogen.org/search>.

“-“ near or below detection level; “+” to “+++” low to highest detected expression for each row. N/A not analysed.

**Table E: CRC risk and Overall survival pM0:** Comparison of the SNPs with p ≤ 0.05 in the Czech discovery set with GWAS results from the Scottish and DACHS replication sets. Amino acid changes are given as <> with the amino acid position indicated. Nominal significance at p ≤ 0.05.

| **Gene** |  | **Czech*** | | | | **Scottish*** | | | | **Czech*** | | | | **DACHS*** | | | |
| --- | --- | --- | --- | --- | --- | --- | --- | --- | --- | --- | --- | --- | --- | --- | --- | --- | --- |
| **SNP** |  | **CRC risk according to genotypes** | | | | | | | | **CRC risk according to allelic probabilities** | | | | | | | |
| **aa change** | **Genotype** | **Cases** | **Controls** | **OR (95%CI)** | **P Val** | **Cases** | **Controls** | **OR (95%CI)** | **P Val** | **Cases** | **Controls** | **OR (95%CI)** | **P Val** | **Cases** | **Controls** | **OR (95%CI)** | **P Val** |
| **NLRP1** | **C/C** | 427 | 284 |  |  | 654 | 2901 |  |  | 1193 | 740 | 1.16 (0.99-1.37) | 0.07 | 1708 | 1707 | 0.96 (0.86-1.08) | 0.51 |
| **rs12150220** | **A/C** | 574 | 355 | 1.08 (0.84-1.39) | 0.56 | 1112 | 4563 | 1.06 (0.95-1.19) | 0.3 |  |  |  |  |  |  |  |  |
| **155: H<>L** | **A/A** | 203 | 108 | 1.41 (1.00-1.99) | **0.05** | 444 | 1786 | 1.05 (0.91-1.22) | 0.48 |  |  |  |  |  |  |  |  |
|  | **A/C + A/A** | 777 | 463 | 1.16 (0.91-1.47) | 0.23 | 1556 | 6349 | 1.06 (0.95-1.18) | 0.29 |  |  |  |  |  |  |  |  |
| **NLRP3** | **C/C** | 1114 | 700 |  |  | - | - |  |  | 1203 | 767 | 0.67 (0.45-1.00) | **0.05** | 1708 | 1707 | 0.95 (0.65-1.41) | 0.82 |
| **rs35829419** | **A/C** | 85 | 66 | 0.63 (0.41-0.97) | **0.04** | - | - | - | - |  |  |  |  |  |  |  |  |
| **705: Q<>K** | **A/A** | 4 | 1 | 0.97 (0.09-10.11) | 0.98 | - | - | - | - |  |  |  |  |  |  |  |  |
|  | **C/A + A/A** | 89 | 67 | 0.64 (0.42-0.98) | **0.04** | - | - | - | - |  |  |  |  |  |  |  |  |
| **NLRP6** | **G/G** | 924 | 629 |  |  | - | - |  |  | 1176 | 757 | 1.16 (1.14-1.17) | **0.04** | 1708 | 1707 | 1 (0.84-1.19) | 0.98 |
| **rs6421985** | **T/G** | 252 | 128 | 1.36 (1.01-1.83) | **0.04** | - | - | - | - |  |  |  |  |  |  | - |  |
| **163: L<>M** | **T/T** | - | - | - | - | - | - | - | - |  |  |  |  |  |  |  |  |
|  | **T/G + T/T** | 252 | 128 | 1.36 (1.01-1.83) | **0.04** | - | - | - | - |  |  |  |  |  |  |  |  |
| **NLRP8** | **G/G** | 732 | 493 |  |  | 1182 | 4939 |  |  | 1210 | 754 | 1.24 (1.01-1.51) | **0.04** | 1708 | 1707 | 1.02 (0.89-1.17) | 0.76 |
| **rs306457** | **C/G** | 415 | 235 | 1.13 (0.88-1.45) | 0.32 | 860 | 3459 | 1.04 (0.94-1.15) | 0.46 |  |  |  |  |  |  |  |  |
| **1049: Ter<>Y** | **C/C** | 63 | 26 | 2.01 (1.09-3.72) | **0.03** | 162 | 690 | 1.01 (0.83-1.23) | 0.88 |  |  |  |  |  |  |  |  |
|  | **C/G + C/C** | 478 | 261 | 1.2 (0.95-1.53) | 0.13 | 1022 | 4149 | 1.04 (0.94-1.14) | 0.49 |  |  |  |  |  |  |  |  |
| **NLRP13** | **C/C** | 419 | 297 |  |  | 753 | 3189 |  |  | 1195 | 756 | 1.26 (1.07-1.49) | **0.01** | 1708 | 1707 | 1.02 (0.92-1.13) | 0.69 |
| **rs303997** | **C/T** | 564 | 334 | 1.37 (1.06-1.77) | **0.02** | 1019 | 4374 | 0.98 (0.88-1.1) | 0.74 |  |  |  |  |  |  |  |  |
| **247: R<>Q** | **T/T** | 212 | 125 | 1.54 (1.10-2.16) | **0.01** | 341 | 1536 | 0.94 (0.81-1.1) | 0.46 |  |  |  |  |  |  |  |  |
|  | **C/T + T/T** | 776 | 459 | 1.42 (1.11-1.80) | **0.005** | 1360 | 5910 | 0.97 (0.87-1.08) | 0.59 |  |  |  |  |  |  |  |  |
| **Gene** |  | **Czech**** | | | | **Scottish**** | | | | **Czech**** | | | | **DACHS**** | | | |
| **SNP** |  | **OS pM0 according to genotypes** | | | | | | | | **OS pM0 according to probabilities** | | | | | | | |
| **aa change** | **Genotype** | **No** | **No died (%)** | **HR (95%CI)** | **p-val** | **No** | **No died (%)** | **HR (95%CI)** | **p-val** | **No** | **No died (%)** | **HR (95%CI)** | **p-val** | **No** | **No died (%)** | **HR (95%CI)** | **p-val** |
| **NLRP1** | **A/A** | 87 | 27 (31.03%) |  |  | 334 | 113 (33.83) |  |  | 293 | 103 (35.15%) | 1.22 (0.93-1.59) | 0.15 | 1406 | 299 (21.27%) | 0.93 (0.79-1.09) | 0.35 |
| **rs12150220** | **A/T** | 142 | 48 (33.80%) | 1.15 (0.71-1.86) | 0.56 | 582 | 173 (29.73) | 0.84 (0.67-1.07) | 0.16 |  |  |  |  |  |  |  |  |
| **155: H<>L** | **T/T** | 64 | 28 (43.75%) | 1.48 (0.87-2.52) | 0.15 | 221 | 71 (32.13) | 0.91 (0.67-1.22) | 0.52 |  |  |  |  |  |  |  |  |
|  | **A/T+T/T** | 206 | 76 (36.89%) | 1.26 (0.80-1.96) | 0.32 | 803 | 244 (30.39) | 0.86 (0.69-1.08) | 0.19 |  |  |  |  |  |  |  |  |
| **NLRP2** | **C/C** | 95 | 39 (41.05%) |  |  | 353 | 118 (33.43) |  |  | 292 | 102 (34.93%) | 0.87 (0.65-1.17) | 0.36 | 1406 | 299 (21.27%) | 1.08 (0.89-1.31) | 0.41 |
| **rs1043673** | **A/C** | 155 | 47 (30.32%) | 0.71 (0.46-1.09) | 0.11 | 589 | 188 (31.92) | 0.97 (0.77-1.22) | 0.76 |  |  |  |  |  |  |  |  |
| **1052: A<>E** | **A/A** | 42 | 16 (38.10%) | 0.87 (0.48-1.56) | 0.63 | 197 | 53 (26.90) | 0.79 (0.57-1.09) | 0.15 |  |  |  |  |  |  |  |  |
|  | **A/C + A/A** | 197 | 63 (31.98%) | 0.74 (0.50-1.11) | 0.15 | 786 | 241 (30.66) | 0.92 (0.74-1.15) | 0.46 |  |  |  |  |  |  |  |  |
| **NLRP5** | **G/G** | 165 | 50 (30.30%) |  |  | 628 | 206 (32.80) |  |  | 293 | 101 (34.47%) | 1.53 (1.12-2.09) | **0.01** | 1406 | 299 (21.27%) | 1.08 (0.84-1.39) | 0.56 |
| **rs10409555** | **A/G** | 109 | 41 (37.61%) | 1.41 (0.93-2.14) | 0.10 | 428 | 132 (30.84) | 0.9 (0.72-1.12) | 0.33 |  |  |  |  |  |  |  |  |
| **1181: V<>I** | **A/A** | 19 | 10 (52.63%) | 2.63 (1.30-5.30) | **0.01** | 80 | 20 (25) | 0.72 (0.46-1.14) | 0.17 |  |  |  |  |  |  |  |  |
|  | **A/G + A/A** | 128 | 51 (39.84%) | 1.54 (1.04-2.29) | **0.03** | 508 | 152 (29.92) | 0.87 (0.7-1.07) | 0.19 |  |  |  |  |  |  |  |  |
| **NLRP5** | **C/C** | 218 | 70 (32.11%) |  |  | 818 | 263 (32.15) |  |  | 295 | 102 (34.58%) | 1.58 (1.07-2.33) | **0.02** | 1406 | 299 (21.27%) | 0.99 (0.77-1.26) | 0.91 |
| **rs12462795** | **C/G** | 73 | 30 (41.10%) | 1.61 (1.03-2.50) | **0.04** | 287 | 84 (29.27) | 0.86 (0.67-1.1) | 0.24 |  |  |  |  |  |  |  |  |
| **1108: S<>C** | **G/G** | 4 | 2 (50.00%) | 2.29 (0.56-9.45) | 0.25 | 24 | 8 (33.33) | 1.09 (0.54-2.22) | 0.80 |  |  |  |  |  |  |  |  |
|  | **C/G + G/G** | 77 | 32 (41.56%) | 1.64 (1.06-2.52) | **0.02** | 311 | 92 (29.59) | 0.88 (0.69-1.11) | 0.28 |  |  |  |  |  |  |  |  |
| **NLRP5** | **T/T** | 209 | 67 (32.06%) |  |  | 787 | 260 (33.04) |  |  | 288 | 101 (35.07%) | 1.3 (0.89-1.88) | 0.17 | 1406 | 299 (21.27%) | 1.15 (0.93-1.43) | 0.18 |
| **rs16986899** | **C/T** | 75 | 34 (45.33%) | 1.62 (1.07-2.45) | **0.02** | 311 | 87 (27.97) | 0.85 (0.66-1.08) | 0.18 |  |  |  |  |  |  |  |  |
| **912: M<>T** | **C/C** | 4 | 0 (0.00%) | 0 (0.00-.) | 0.98 | 39 | 11 (28.21) | 0.79 (0.43-1.45) | 0.45 |  |  |  |  |  |  |  |  |
|  | **C/T + C/C** | 79 | 34 (43.04%) | 1.5 (0.99-2.27) | 0.06 | 350 | 98 (28) | 0.84 (0.67-1.06) | 0.14 |  |  |  |  |  |  |  |  |
| **NLRP12** | **C/C** | 182 | 70 (38.46%) |  |  | 697 | 210 (30.13) |  |  | 284 | 97 (34.15%) | 0.71 (0.49-1.04) | 0.08 | 1406 | 299 (21.27%) | 0.88 (0.63-1.23) | 0.46 |
| **rs34436714** | **A/C** | 88 | 23 (26.14%) | 0.64 (0.40-1.04) | 0.07 | 384 | 131 (34.11) | 1.14 (0.91-1.41) | 0.26 |  |  |  |  |  |  |  |  |
| **42: K<>N** | **A/A** | 14 | 4 (28.57%) | 0.67 (0.24-1.85) | 0.44 | 58 | 18 (31.03) | 0.98 (0.61-1.59) | 0.93 |  |  |  |  |  |  |  |  |
|  | **A/C + A/A** | 102 | 27 (26.47%) | 0.65 (0.41-1.01) | 0.06 | 442 | 149 (33.71) | 1.11 (0.9-1.37) | 0.32 |  |  |  |  |  |  |  |  |
| **NLRC5** | **C/C** | 150 | 61 (40.67%) |  |  | 596 | 178 (29.87) |  |  | 298 | 104 (34.90%) | 0.79 (0.58-1.09) | 0.16 | 1406 | 299 (21.27%) | 1.12 (0.94-1.33) | 0.22 |
| **rs289723** | **A/C** | 122 | 34 (27.87%) | 0.65 (0.42-0.99) | **0.04** | 456 | 147 (32.24) | 1.12 (0.9-1.39) | 0.32 |  |  |  |  |  |  |  |  |
| **1105: Q<>K** | **A/A** | 26 | 9 (34.62%) | 0.88 (0.44-1.77) | 0.72 | 85 | 33 (38.82) | 1.27 (0.87-1.84) | 0.21 |  |  |  |  |  |  |  |  |
|  | **A/C + A/A** | 148 | 43 (29.05%) | 0.68 (0.46-1.01) | **0.06** | 541 | 180 (33.27) | 1.14 (0.93-1.4) | 0.21 |  |  |  |  |  |  |  |  |
| **NLRC5** | **C/C** | 225 | 72 (32.00%) |  |  | - | - | - | - | 297 | 105 (35.35%) | 1.61 (1.12-2.31) | **0.01** | 1412 | 297 (21.03%) | 0.77 (0.61-0.97) | **0.03** |
| **rs74439742** | **C/T** | 65 | 29 (44.62%) | 1.57 (1.01-2.43) | **0.04** | - | - | - | **-** |  |  |  |  |  |  |  |  |
| **191: P<>L** | **T/T** | 7 | 4 (57.14%) | 2.82 (1.00-7.96) | 0.05 | - | - | - | - |  |  |  |  |  |  |  |  |
|  | **C/T + T/T** | 72 | 33 (45.83%) | 1.65 (1.08-2.51) | **0.02** | - | - | - | **-** |  |  |  |  |  |  |  |  |
